# A crucial role of the malate aspartate shuttle in metabolic reprogramming in TNF-induced SIRS

**DOI:** 10.3389/fimmu.2025.1652516

**Published:** 2025-10-08

**Authors:** Louise Nuyttens, Marah Heyerick, Maxime Roes, Elise Moens, Céline Van Dender, Charlotte Wallaeys, Tino Hochepied, Steven Timmermans, Jolien Vandewalle, Claude Libert

**Affiliations:** ^1^ Center for Inflammation Research, Vlaams Instituut Voor Biotechnologie (VIB), Ghent, Belgium; ^2^ Department of Biomedical Molecular Biology, Ghent University, Ghent, Belgium

**Keywords:** malate aspartate shuttle, citrin, TNF-induced SIRS, lipid metabolism, carbohydrate metabolism

## Abstract

Tumor necrosis factor (TNF) causes a lethal systemic inflammatory response syndrome (SIRS) which is characterized by significant metabolic alterations. Based on liver RNA sequencing, we found that TNF impairs the malate-aspartate shuttle (MAS), an essential redox shuttle that transfers reducing equivalents across the inner mitochondrial membrane thereby recycling cytosolic NAD^+^. This downregulation of MAS genes in TNF-induced SIRS likely results from loss of HNF4α function, which appears to be the key transcription factor involved. Using Slc25a13^-/-^ mice lacking citrin – a crucial MAS component – we demonstrate that MAS dysfunction exacerbates TNF-induced metabolic dysregulations and lethality. Disruptive NAD^+^ regeneration leads to diminished mitochondrial β-oxidation, leading to elevated levels of circulating free fatty acids (FFAs) and to hepatic lipid accumulation. Simultaneously, MAS dysfunction promotes glycolysis coupled to lactate production and reduces lactate-mediated gluconeogenesis, culminating in severe hyperlactatemia that triggers VEGF-induced vascular leakage. Overall, MAS dysfunction contributes to metabolic failure and lethality in TNF-induced SIRS, highlighting its potential as a promising, therapeutic target.

## Introduction

Systemic inflammatory response syndrome (SIRS) is marked by an uncontrolled and unbalanced inflammatory response caused by a variety of sterile triggers (e.g. burns, trauma or acute pancreatitis) or upon infection (1, 2). It is associated with a ‘cytokine storm’ in the circulation, involving the secretion of pro-inflammatory cytokines such as tumor necrosis factor (TNF) (3).

TNF is a multifunctional, pro-inflammatory cytokine involved in a plethora of biological processes, including immune function, cellular proliferation, cell death and energy metabolism, as well as in diseases such as inflammatory bowel disease and rheumatoid arthritis (4–6). TNF signaling is mediated through binding and activation of two different transmembrane receptors, TNF receptor 1 (TNFR1 or p55) and TNF receptor 2 (TNFR2 or p75), with TNFR1 being primarily responsible for TNF’s versatile proinflammatory effects via AP1 or NF-κB pathway activation (7). As one of the first cytokines discovered, and based on its impressive anticancer effect, TNF biology has been studied very intensely (8). Yet, key factors involved in the development of SIRS by TNF remain to be discovered. Exogenous TNF administration is a well-known experimental model to induce lethal SIRS as it triggers an acute inflammatory response marked by excessive release of several toxic mediators such as interleukins (e.g. IL-1, IL-6, IL-17), type I interferons and matrix metalloproteinases (3, 9–13). TNF-induced lethal SIRS is characterized by hypothermia, hypotension, tissue damage, multiple organ failure and significant metabolic reprogramming, which is similarly observed in human patients following surgery, trauma, infection, burns or pancreatitis (3, 9).

Studies have shown that TNF signaling has a prominent effect on carbohydrate and lipid metabolism. Many *in vitro* studies demonstrate that TNF treatment diminishes oxidative metabolism and mitochondrial function while inducing glycolytic flux, leading to a shift towards a Warburg-like metabolism in, for instance, epithelial cells, fibroblasts, hepatocytes and skeletal muscle cells (14–19). This is characterized by increased glycogenolysis and glucose consumption, upregulated expression of glycolytic enzymes, lactate secretion, elevated glycolytic ATP production and decreased mitochondrial oxygen consumption. Similar effects have been observed in *in vivo* studies, confirming that TNF signaling plays a crucial role in shifting towards glycolytic metabolism in the liver, spleen and monocytic cells of mice upon infection (18, 20).

TNF signaling has also an impact on lipid metabolism (6, 21, 22). TNF reduces fatty acid uptake and lipogenesis, but it promotes lipolysis in human and rodent adipocytes leading to the release and accumulation of circulating free fatty acids (FFAs) and glycerol (23–27). However, the conversion of FFA into acetyl-CoA via mitochondrial FFA β-oxidation is severely impaired by TNF in epithelial cells or isolated hepatocytes (28–31). These disruptions contribute to dyslipidemia resulting in severe complications such as ectopic lipid accumulation (6). Indeed, many studies highlight an important role of TNF in hepatic steatosis and lipotoxicity as blocking TNF signaling via TNFR1 significantly improved fat accumulation in mouse models of metabolic dysfunction-associated steatotic liver disease (32–34).

In all cells, adequate levels of NAD^+^ and NADH are essential for maintaining energy metabolism during homeostasis (35, 36). Metabolism involves cellular oxidation reactions (e.g. glycolysis, TCA cycle, β-oxidation), in which NAD^+^ serves as an electron acceptor, resulting in the production of NADH. The formed NADH transfers these electrons to the mitochondrial electron transport chain (ETC), on the one hand driving ATP synthesis, and on the other hand regenerating NAD^+^. Since the inner mitochondrial membrane is impermeable to NADH, this electron transfer from cytosolic NADH to the mitochondrial matrix is mediated by NAD^+^/NADH redox shuttles, i.e. the malate-aspartate shuttle (MAS) and the glycerol-3-phosphate shuttle. The MAS is the primary redox shuttle and consists of four enzymes: (1) cytosolic and (2) mitochondrial malate dehydrogenase 1/2 (resp. MDH1 & MDH2), (3) cytosolic and (4) mitochondrial aspartate aminotransferase (resp. GOT1 & GOT2), and two mitochondrial transporters: the oxoglutarate (i.e. α-ketoglutarate)-malate carrier (OGC; SLC25A11) and the aspartate-glutamate carrier (AGC; SLC25A13), also known as citrin (37). MAS activity results in both cytosolic NAD^+^ and mitochondrial NADH regeneration needed for the continuation of cytosolic oxidative pathways (e.g. glycolysis) and for maintaining mitochondrial oxidative phosphorylation, respectively (37–40). Impairment of MAS activity could therefore lead to profound metabolic alterations. For instance, citrin deficiency is a rare autosomal recessive metabolic disease caused by mutations in the *Slc25a13* gene. Citrin is located in the inner mitochondrial membrane and is responsible for cytosolic glutamate import while simultaneously exporting aspartate from the mitochondria (41, 42). Patients with citrin deficiency exhibit a MAS disruption affecting metabolic pathways including glycolysis, gluconeogenesis (GNEO), lipid metabolism and the TCA cycle, resulting in hypoglycemia, dyslipidemia, hepatic steatosis and an energy deficit (43). However, the presence of MAS inactivity and its potential crosstalk with metabolic dysregulations in TNF-induced SIRS remains to be explored.

As one of the major hepatocyte identity-determining transcription factors, hepatocyte nuclear factor alpha (HNF4α) is involved in the transcription of numerous genes and HNF4α was found to lose function during metabolic diseases and sepsis, and is related to several metabolic abnormalities, such as FFA-induced steatosis (44, 45).

We report that TNF causes an impaired MAS activity, which might result from HNF4α loss-of-function and contributes to profound metabolic alterations in TNF-induced SIRS. To investigate this in more detail, we utilized Slc25a13^-/-^ mice, which lack citrin, an essential component of the MAS. Our findings demonstrate that citrin loss-of-function exacerbates TNF-induced metabolic dysfunctions and lethality by depleting NAD^+^ and NADH levels. This results in (1) impaired mitochondrial β-oxidation leading to elevated FFA levels and ectopic lipid droplet formation, and (2) enhanced glycolysis combined with more lactate production and diminished lactate-mediated gluconeogenesis, resulting in severe hyperlactatemia driving VEGF-mediated vascular permeability. Together, these metabolic disruptions contribute to lethality in TNF-induced SIRS. Our data thus unfold a previously unrecognized important pathophysiological change, induced by TNF and directly related to its lethal nature.

## Results

### TNF-induced SIRS is characterized by significant metabolic reprogramming

**Figure 1:**
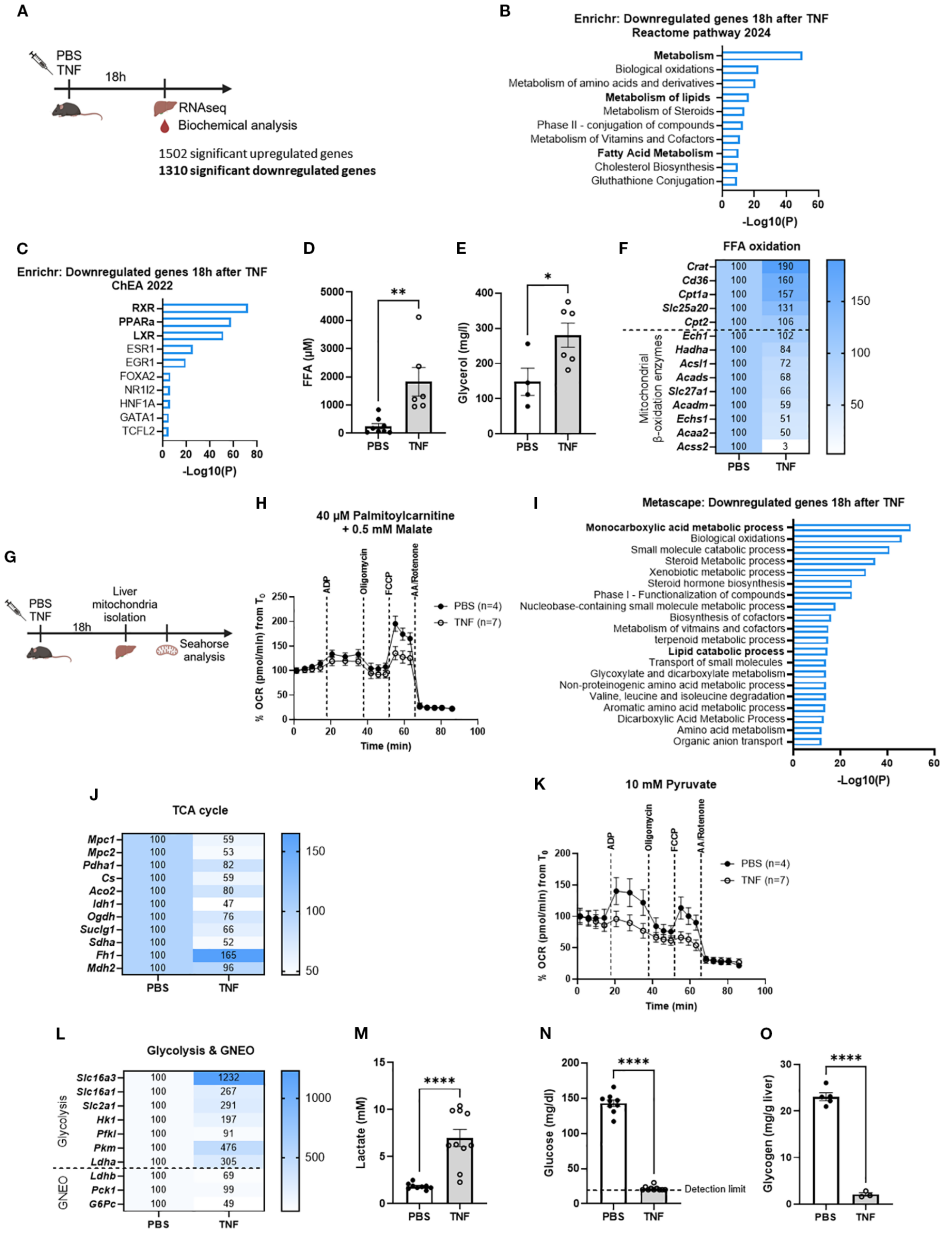
TNF-induced SIRS is characterized by significant metabolic reprogramming. **(A)**. Experimental setup for liver genome-wide transcriptomic analysis 18h after TNF (25 µg)**. (B, C)**. Enrichr pathway analysis (Reactome pathway 2024 and ChEA 2022) of the downregulated genes 18h after TNF (1310 genes) with LFC < -1 and p < 0.05. **(D, E)**. Plasma FFA **(D)** and glycerol **(E)** levels 18h after TNF (35 µg). n=4-8/group. **(F)**. Heatmap depicting the relative expression levels (%) of FFA oxidation genes 18h after TNF, with RNASeq mRNA counts from the PBS condition set at 100%. **(G)**. Experimental setup for seahorse analysis. **(H)**. OCR flux of isolated liver mitochondria of PBS- and TNF-treated mice (35 µg), driven by 40 µM palmitoylcarnitine and 0.5 mM malate. n=4-7/group. **(I)**. Metascape pathway analysis of the downregulated genes 18h after TNF (1310 genes) with LFC < -1 and p < 0.05. **(J)**. Heatmap depicting the relative expression levels (%) of TCA cycle genes 18h after TNF, with RNASeq mRNA counts from the PBS condition set at 100% **(K)**. OCR flux of isolated liver mitochondria of PBS- and TNF-treated mice, driven by 10 mM pyruvate. n=4-7/group. **(L)**. Heatmap depicting the relative expression levels (%) of glycolysis and gluconeogenesis (GNEO) genes 18h after TNF, with RNASeq mRNA counts from the PBS condition set at 100%. **(M–O)**. Blood lactate **(M)**, blood glucose **(N)** and liver glycogen **(O)** levels 18h after TNF (35 µg). n=3-10/group. Bars: mean ± SEM. Each dot represents a single biological replicate. P-values were analyzed with unpaired t-test **(D, E, M–O)**. ****p ≤ 0.0001,**p ≤ 0.01, *p ≤ 0.05.

To corroborate the presence of metabolic alterations in TNF-induced SIRS, functional enrichment analyses were performed on a liver bulk RNA sequencing (RNASeq) dataset retrieved 18h after injection of a lethal TNF dose in mice (viz. 25 µg), i.e. during the acute phase of TNF-induced SIRS (46). TNF challenge resulted in 1502 upregulated genes (log fold change (LFC) > 1 and p < 0.05) and 1310 downregulated genes (LFC < -1 and p < 0.05) (**Figure 1A**). As expected, Enrichr and Metascape analyses of the upregulated genes demonstrated a clear enrichment related to pro-inflammatory transcription factors and immune responses (**Supplementary Figures S1A–D**). Notably, Enrichr analysis of the downregulated genes identified metabolism as the top hit, clearly suggesting the presence of impaired metabolic processes in TNF-induced SIRS (**Figure 1B**).

Further analysis specifically exhibited a significant downregulation of lipid metabolism, as indicated by the association of downregulated genes with retinoid X receptor (RXR), peroxisome proliferator-activated receptor alpha (PPARα) and liver X receptor (LXR), three transcription factors involved in regulating lipid metabolism (**Figure 1C**), and their enrichment for pathways like metabolism of lipids and fatty acid metabolism (**Figure 1B**). To validate these transcriptome findings, we measured several lipid metabolic parameters 18h after TNF injection (**Figure 1A**). Both plasma FFA and glycerol levels were significantly elevated in TNF-treated mice compared to the PBS controls, suggesting the presence of increased white adipose tissue (WAT) lipolysis and/or reduced FFA oxidation (**Figures 1D, E**). However, no significant decrease in body weight nor inguinal WAT (iWAT) weight of TNF-treated mice could be observed (**Supplementary Figures S1E, F**), implying that the normally observed TNF-induced lipolysis is not markedly activated at that time point or is too limited to be detected by weight of the WAT (23–25). Examination of the relative expression levels of genes involved in FFA oxidation revealed a surprising upregulation of genes specifically associated with fatty acid uptake and mitochondrial import (e.g. *Cd36* and *Slc25a20*), whereas mitochondrial FFA β-oxidation genes (e.g. *Acsl1*) were significantly downregulated upon TNF challenge (**Figure 1A**). This suggests a potential impairment in FFA oxidation, particularly at the mitochondrial β-oxidation level. We next performed Seahorse analysis to assess the FFA β-oxidation capacity of isolated liver mitochondria from PBS- and TNF-treated mice (**Figure 1G**). Palmitoylcarnitine-driven oxygen consumption rate (OCR) in TNF mitochondria was severely impaired compared to PBS mitochondria (**Figure 1H**, **Supplementary Figure S1G**). These findings support that TNF-induced SIRS is characterized by compromised fatty acid metabolism, reflected by diminished FFA β-oxidation leading to elevated FFA levels.

To affirm the Enrichr results, we next performed Metascape analysis on the downregulated genes, which interestingly identified monocarboxylic acid metabolic process as the top enriched term (**Figure 1I**). This encompasses all chemical reactions and pathways involving monocarboxylic acids, including subprocesses such as fatty acid metabolism, but also pyruvate and lactate metabolism. This potentially suggests a disruption in carbohydrate metabolism, consistent with previous studies showing the presence of Warburg-like metabolism upon TNF treatment (14, 18, 19). We substantiate this finding, noting significantly diminished relative expression levels of genes involved in pyruvate-driven TCA cycle capacity and demonstrating reduced pyruvate-driven OCR in mitochondria after a lethal TNF dose (**Figures 1A, J, K**, **Supplementary Figure S1H**). This goes along with the presence of an increased glycolytic flux, suggested by enhanced relative expression levels of glycolytic genes upon TNF challenge (**Figures 1A, L**). This was further supported by the presence of profound hyperlactatemia, hypoglycemia and depleted liver glycogen stores, proving enhanced glucose utilization through glycolysis after a lethal TNF dose (**Figures 1A, M–O**). Additionally, the relative expression levels of genes encoding enzymes in the lactate-mediated GNEO pathway (*Ldhb*, *Pck1* and *G6Pc*) were significantly diminished upon TNF injection (**Figures 1A, L**). This suggests that the observed severe hypoglycemia and hyperlactatemia are not only driven by increased glucose consumption and lactate production via glycolysis, but also by impaired glucose production and lactate usage through the GNEO pathway.

**Figure 2:**
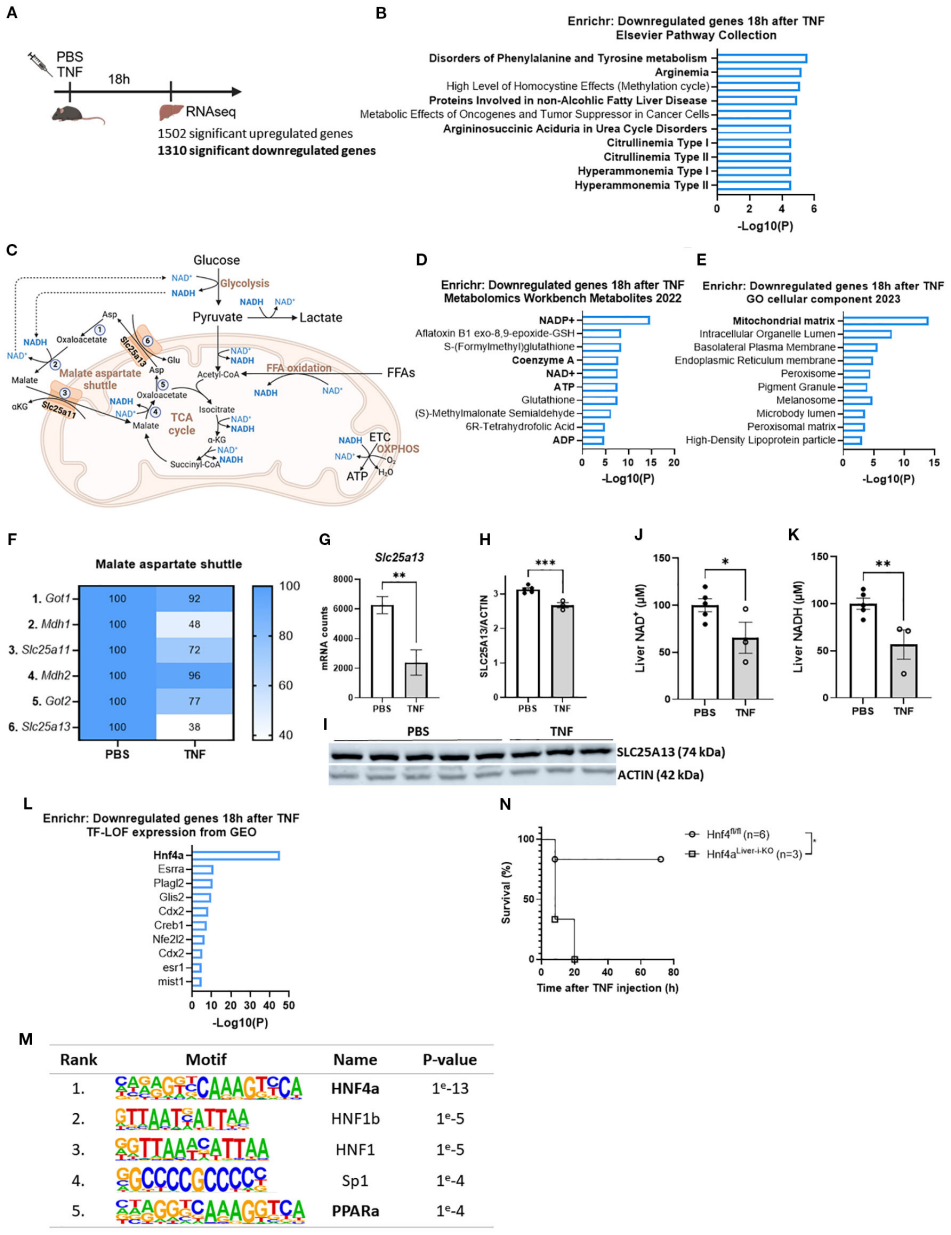
TNF impairs the malate aspartate shuttle leading to a NAD^+^ and NADH deficit. **(A)** Experimental setup for liver genome-wide transcriptomic analysis 18h after TNF (25 µg). **(B)** Enrichr pathway analysis (Elsevier pathway collection) of the downregulated genes 18h after TNF (1310 genes) with LFC < -1 and p < 0.05. **(C)** Schematic overview of the MAS and metabolic pathways depending on NAD^+^/NADH homeostasis. 1: GOT1; 2: MDH1; 3. OGC; 4: MDH2; 5: GOT2; 6: AGC; ETC: electron transport chain; OXPHOS: oxidative phosphorylation; αKG: alpha ketoglutarate. **(D, E)**. Enrichr pathway analysis (Metabolomics Workbench metabolites 2022 **(D)** and GO cellular component 2023 **(E)** of the downregulated genes 18h after TNF (1310 genes) with LFC < -1 and p < 0.05. **(F)** Heatmap depicting the relative expression levels (%) of genes involved in the MAS 18h after TNF, with RNASeq mRNA counts from the PBS condition set at 100%. **(G)** mRNA counts of *Slc25a13* in livers of PBS- and TNF-treated mice. n=3/group. **(H, I)**. Western blot analysis of SLC25A13 (74 kDa) protein levels in livers of PBS- and TNF-treated mice (35 µg), normalized to actin levels (42 kDa). n=3-5/group. **(J, K)**. Liver NAD^+^ **(J)** and NADH **(K)** levels in PBS- and TNF-injected mice (35 µg), relative to PBS controls. n=3-5/group. **(L)**. Enrichr pathway analysis (TF-LOF expression from GEO) of the downregulated genes 18h after TNF (1310 genes) with LFC < -1 and p < 0.05. **(M)**. HOMER motif analysis of the 1310 downregulated genes. **(N)**. Hnf4α^Liver-i-KO^ and Hnf4α^fl/fl^ were IP injected with TNF (32,5 µg) and mortality was monitored. n=3-6/group. Bars: mean ± SEM. Each dot represents a single biological replicate. P-values were analyzed with unpaired t-test **(G, H, J, K)**. Survival was analyzed via a log rank test **(N)**. **p ≤ 0.01, *p ≤ 0.05.

Overall, TNF-induced SIRS is marked by profound metabolic reprogramming characterized by diminished mitochondrial FFA β-oxidation, increased glycolysis, impaired GNEO and reduced mitochondrial respiration.

### TNF impairs the malate aspartate shuttle leading to a NAD^+^ and NADH deficit

To identify a potential, coherent mechanism underlying all metabolic disturbances in TNF-induced SIRS, we focused on the ‘Elsevier Pathway Collection’ in Enrichr for TNF-downregulated genes (1310, LFC < -1 and p < 0.05). Remarkably, this revealed a clear hit for citrullinemia (**Figures 2A, B**). Citrullinemia is a rare autosomal recessive urea cycle disorder caused by mutations in either ASS1 (type 1), encoding argininosuccinate synthetase which is an urea cycle enzyme, or SLC25A13 (type 2, also called citrin deficiency), encoding the aspartate-glutamate carrier (citrin) which results in insufficient aspartate supply to the urea cycle (47). Since citrin is a crucial component of the MAS, citrin deficiency is also characterized by impaired MAS activity, disrupting the cytosolic and mitochondrial NAD^+^ and NADH levels and affecting multiple NAD^+^/NADH dependent metabolic pathways, including glycolysis, GNEO, TCA cycle and FFA β-oxidation (**Figure 2C**), all pathways that we found to be significantly affected in TNF-induced SIRS (**Figure 1**) (43).

With this, further Enrichr analyses displayed that the TNF-downregulated genes (1310, LFC < -1 and p < 0.05) were also related to key metabolites, including NADP^+^, NAD^+^, ATP and ADP, coenzyme A, and to the mitochondrial matrix as the most affected cellular component (**Figures 2D, E**). This implies diminished NADP^+^ and NAD^+^ levels and impaired mitochondrial energy homeostasis. Combined with the association of the downregulated genes with citrullinemia, this proposes the presence of citrin deficiency leading to MAS inactivity and disrupted NAD^+^ and NADH levels. Indeed, the relative expression levels of the MAS genes were markedly diminished, with *Slc25a13* showing the most significant downregulation upon TNF challenge (**Figures 2F, G**). This was associated with significantly diminished liver SLC25A13 and SLC25A11 protein levels (**Figures 2H, I**, **Supplementary Figures S2A–C**). Moreover, NAD^+^ and NADH levels in total liver lysates were significantly diminished after a lethal TNF dose (**Figures 2J, K**). Since the cytosolic fraction contains significantly more NAD^+^ of the total NAD^+^ pool and the mitochondrial fraction contains significantly more NADH of the total NADH pool, these reductions likely reflect diminished cytosolic NAD^+^ and mitochondrial NADH levels. Hence, these findings demonstrate that TNF-induced SIRS is characterized by impaired MAS activity resulting in a deficit of NAD^+^ and NADH, which eventually could lead to metabolic disturbances.

On the other hand, to identify the mechanism underlying *Slc25a13* downregulation in TNF-induced SIRS, we focused on the ‘transcription factor – loss of function (LOF)’ dataset in Enrichr for TNF-downregulated genes (1310, LFC < -1 and p < 0.05). This identified HNF4α, a key nuclear transcription factor, as the top hit (**Figure 2L**), very far beyond other factors. Moreover, the promoter regions of these downregulated genes were significantly more enriched for the HNF4α motif (**Figure 2M**). This clearly shows the presence of HNF4α LOF in TNF-induced SIRS. Moreover, hepatocyte-specific HNF4α knockout (Hnf4α^Liver-i-KO^) mice were significantly more sensitive to a TNF dose that was sublethal in Hnf4α^fl/fl^ (control) mice (**Figure 2N**), demonstrating the crucial role of hepatic HNF4α in surviving TNF-induced SIRS. Interestingly, *Slc25a13* is among the genes associated with HNF4α LOF, and GeneCards indicates that its promoter region contains a HNF4α binding site. This was confirmed by HNF4α CHIP-Seq identifying a distinct HNF4α peak in the *Slc25a13* promoter region (**Supplementary Figure S2D**). Additionally, exploring the liver mRNA counts of *Slc25a13* in a previously published liver Hnf4α^Liver-i-KO^ dataset (GSE260635; Van Dender et al. (44)) showed significantly reduced liver *Slc25a13* mRNA counts in Hnf4α^Liver-i-KO^ livers compared to Hnf4α^fl/fl^ mouse livers proving that *Slc25a13* expression is HNF4α dependent (**Supplementary Figure S2E**). Hence, this strongly suggests that *Slc25a13* downregulation is mediated by HNF4α LOF in TNF-induced SIRS.

### Citrin knockout results in acute lethality and enhanced metabolic dysfunctions in TNF-induced SIRS

**Figure 3:**
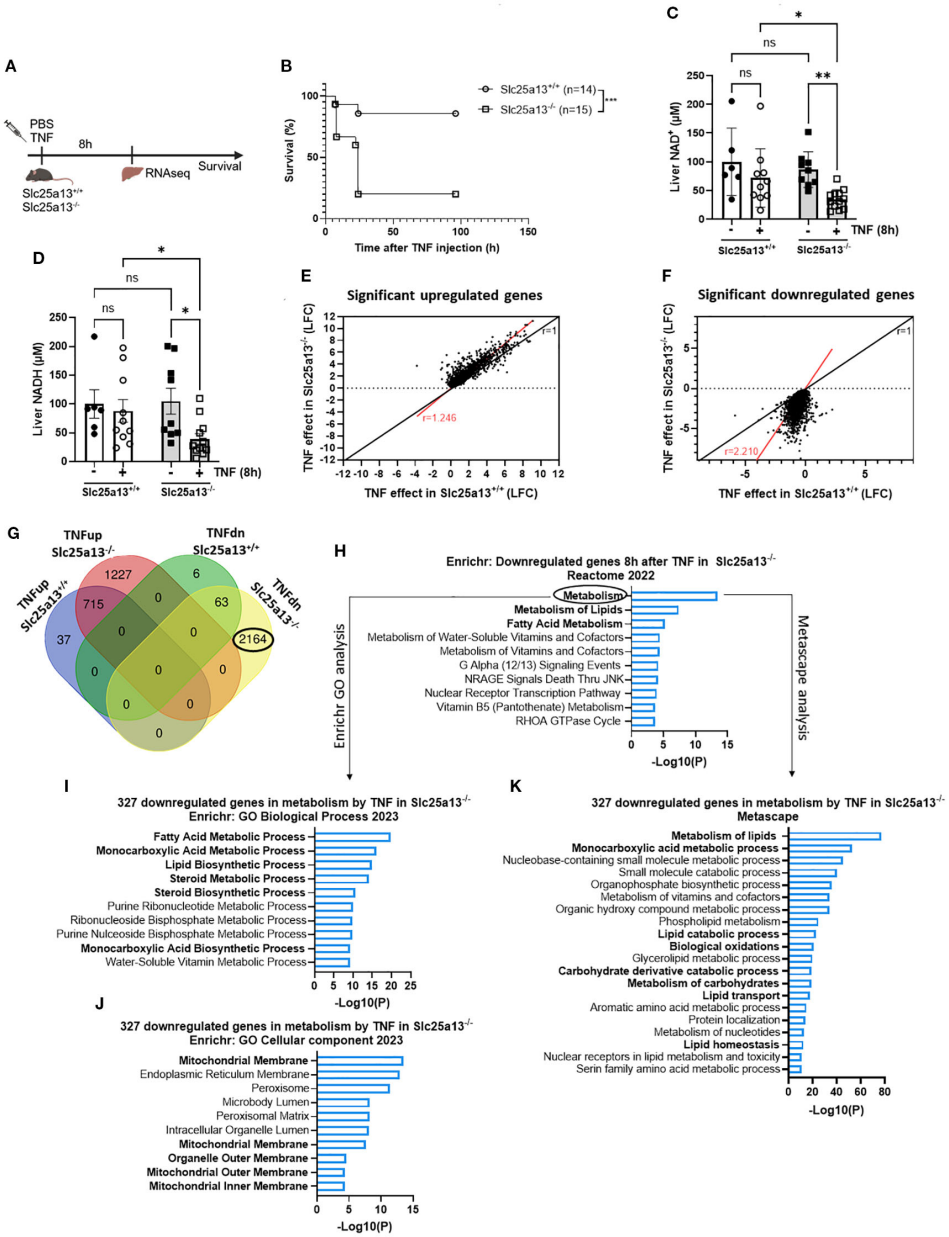
Citrin knockout results in acute lethality and enhanced metabolic dysfunctions in TNF-induced SIRS. **(A)** Experimental setup for liver genome-wide transcriptomic analysis and survival after TNF (35 µg). **(B)** Slc25a13^+/+^ and Slc25a13^-/-^ were IP injected with TNF (35 µg) and mortality was monitored. n=14-15/group. **(C, D)**. Liver NAD^+^ **(C)** and NADH **(D)** levels in PBS- and TNF-treated Slc25a13^+/+^ and Slc25a13^-/-^ mice, relative to PBS wildtype controls. n=6-12/group. **(E, F)**. Scatter plot depicting the log fold changes (LFC) of differentially upregulated **(E)** or downregulated **(F)** genes in TNF-treated Slc25a13^-/-^ mice compared to TNF-treated Slc25a13^+/+^ mice. The black line represents the diagonal (r=1) and the red line indicates the real slope [r=1.246 **(E)** or r=2.210 **(F)**] of the data. Data were analyzed with linear regression. **(G)** Venn diagram depicting the number and overlap of up- and downregulated genes (LFC > 1 or LFC < -1 and p < 0.05) by TNF in Slc25a13^+/+^ and Slc25a13^-/-^ mice. **(H)** Enrichr pathway analysis (Reactome 2022) of the unique downregulated genes 8h after TNF (2164 genes) in Slc25a13^-/-^ mice with LFC < -1 and p < 0.05. **(I–K)**. Enrichr pathway analysis (GO Biological Process 2023 **(I)** and GO Cellular component 2023 **(J)**) and Metascape pathway analysis **(K)** of the downregulated genes associated with metabolism 8h after TNF (327 genes) with LFC < -1 and p < 0.05. Bars: mean ± SEM. Each dot represents a single biological replicate. P-values were analyzed with two-way ANOVA **(C, D)**. Survival was analyzed via a log rank test **(B)**. ***p ≤ 0.001, **p ≤ 0.01, *p ≤ 0.05, ns, not significant.

To investigate the potential role of MAS dysfunction in metabolic reprogramming and lethality in TNF-induced SIRS, we generated Slc25a13^-/-^ mice. Full knockout validation was performed by measuring liver SLC25A13 protein levels via western blotting, confirming the total absence of SLC25A13 protein in Slc25a13^-/-^ mice (**Supplementary Figures S3A, B**). Strikingly, Slc25a13^-/-^ mice showed acute lethality in response to a TNF dose that was sublethal in Slc25a13^+/+^ mice (**Figures 3A, B**). This was accompanied by more pronounced hypothermia and hyperlactatemia over time in Slc25a13^-/-^ mice after TNF injection (**Supplementary Figures S3C, D**). Moreover, both NAD^+^ and NADH levels in total liver lysates were already significantly reduced in Slc25a13^-/-^ mice 8h post-TNF injection, a difference that was not yet detected in Slc25a13^+/+^ mice (**Figures 3C, D**). Interestingly, no basal differences in liver NAD^+^ and NADH levels are notable between Slc25a13^+/+^ and Slc25a13^-/-^ mice as during homeostasis this is compensated by glycerol-3-phosphate shuttle activity, as evidenced by other studies (48, 49). Overall, this shows that citrin deficiency impairs MAS activity leading to a more rapid NAD^+^ and NADH depletion in TNF-induced SIRS, and more importantly, this emphasizes a crucial role of MAS in resisting TNF lethality.

To examine the mechanisms underlying the increased TNF sensitivity of Slc25a13^-/-^ mice, RNASeq was performed on livers of Slc25a13^+/+^ and Slc25a13^-/-^ mice 8h after PBS or TNF injection, a timepoint chosen based on the NAD^+^ and NADH effects (**Figure 3A**). Citrin deficiency alone resulted in only four significantly differentially expressed genes (*Slc25a13*, *Map1a*, *Asns* and *Zbed*), indicating no notable baseline differences between Slc25a13^+/+^ and Slc25a13^-/-^ mice. When plotting the log fold changes (LFCs) of all significantly upregulated or downregulated genes (p < 0.05) in both Slc25a13^+/+^ and Slc25a13^-/-^ mice upon TNF challenge, a clearly more pronounced transcriptional response to TNF was observed in Slc25a13^-/-^ mice compared to Slc25a13^+/+^ mice, especially for the downregulated genes (Upregulated genes: **Figure 3E**, red line slope = 1.246; downregulated genes: **Figure 3F**, red line slope = 2.210). We detected 752 and 1942 significantly upregulated genes and 69 and 2227 significantly downregulated genes (LFC > 1 or < -1 and p < 0.05) by TNF in Slc25a13^+/+^ and Slc25a13^-/-^ mice, respectively (**Figure 3G**). Hence, TNF caused a substantially stronger transcriptional response in Slc25a13^-/-^ mice, with a 2.6- and 33-fold increase in the number of up- and downregulated genes, respectively, compared to Slc25a13^+/+^ mice.

Enrichr analysis of the 1227 uniquely upregulated genes by TNF in Slc25a13^-/-^ mice demonstrated a clear enrichment in pro-inflammatory responses (**Supplementary Figure S3E**). More specifically, heatmap depiction of the LFCs of genes involved in inflammation and endothelial activation demonstrated a stronger upregulation in Slc25a13^-/-^ mice compared to Slc25a13^+/+^ mice 8h post-TNF injection (**Supplementary Figure S3F**). These results imply that citrin deficiency enhances the transcriptional inflammatory response upon TNF stimulation. However, the difference in differential gene expression upon TNF injection was much more pronounced for the downregulated genes compared to the upregulated genes between Slc25a13^+/+^ and Slc25a13^-/-^ mice (**Figures 3E, F**). Therefore, we focused on the uniquely 2164 significantly downregulated genes by TNF in Slc25a13^-/-^ mice to explore potential mechanisms contributing to their increased TNF sensitivity. Interestingly, Enrichr analysis of these downregulated genes revealed metabolism as the top hit, followed by ‘metabolism of lipids’ and ‘fatty acid metabolism’ (**Figure 3H**). Transcription factor analysis (ChEA 2022) also revealed a clear enrichment for RXR, PPARα and LXR, clearly suggesting a significant downregulation of lipid metabolism (**Supplementary Figure S3G**). We next performed further functional analyses on the 327 downregulated genes specifically associated with metabolism. Both Enrichr and Metascape analyses revealed a clear hit for lipid, fatty acid and monocarboxylic acid metabolic processes, and the mitochondrial membrane as the most affected cellular component (**Figures 3I–K**). Clearly, these functional enrichment analyses 8h after TNF injection in Slc25a13^-/-^ mice closely resembled the functional enrichment analyses observed 18h after lethal TNF injection in Slc25a13^+/+^ mice (**Figure 1**). When plotting the LFCs of all differentially expressed genes (p < 0.05) in Slc25a13^+/+^ upon 18h TNF treatment versus their LFC in Slc25a13^-/-^ mice upon 8h TNF treatment, a similar transcriptional signature response to TNF was observed (**Supplementary Figure S3H**, red line slope = 0.7814 versus black diagonal). Hence, these data strongly suggest that citrin LOF and subsequent MAS inactivity play a crucial role in the development of metabolic dysregulations, i.e. fatty acid and carbohydrate metabolism, and that this contributes to lethality in TNF-induced SIRS.

### Citrin knockout worsens lipid metabolic dysfunctions in TNF-induced SIRS

**Figure 4:**
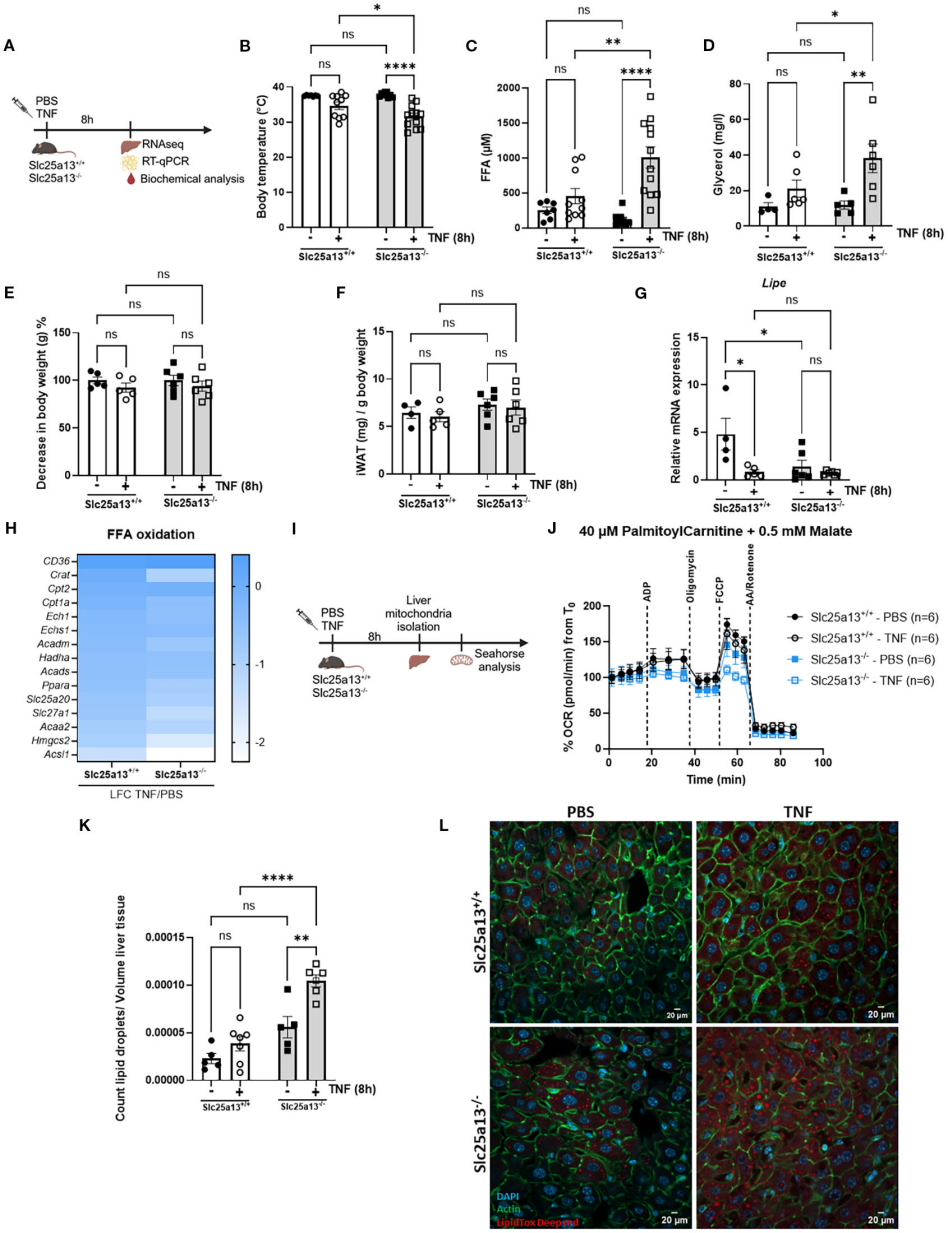
Citrin knockout worsens lipid metabolic dysfunctions in TNF-induced SIRS. **(A)** Experimental setup 8h after TNF (35 µg). **(B–D)** Body temperature **(B)**, plasma FFA **(C)** and glycerol **(D)** levels in PBS- and TNF-treated Slc25a13^+/+^ and Slc25a13^-/-^ mice. n=4-12/group. **(E)** % decrease in body weight of PBS- and TNF-treated Slc25a13^+/+^ and Slc25a13^-/-^ mice, with body weight from the PBS condition set at 100%. n=5-6/group. **(F)** Weight of the inguinal fat pad (iWAT) relative to the total body weight of PBS- and TNF-treated Slc25a13^+/+^ and Slc25a13^-/-^ mice. n=4-6/group. **(G)** *Lipe* mRNA expression in livers of PBS- and TNF-treated Slc25a13^+/+^ and Slc25a13^-/-^ mice, relative to *Gapdh* and *Tbp.* n=4-6/group. **(H)** Heatmap depicting the LFC (TNF versus PBS) of FFA oxidation genes in Slc25a13^+/+^ and Slc25a13^-/-^ mice. **(I)** Experimental setup for Seahorse analysis. **(J)** OCR flux of isolated liver mitochondria of PBS- and TNF-treated Slc25a13^+/+^ and Slc25a13^-/-^ mice, driven by 40 µM palmitoylcarnitine and 0.5 mM malate. n=6/group. **(K, L)** Count of lipid droplets relative to liver tissue volume was determined for each Z-stack and averaged over all Z-stack liver sections of PBS- and TNF treated Slc25a13^+/+^ and Slc25a13^-/-^ mice **(K)**. Immunofluorescent images of liver of PBS- and TNF treated Slc25a13^+/+^ and Slc25a13^-/-^ mice stained with Acti-stain (green), DAPI (blue) and LipidTOX (red). Z-stacks were generated in 8 regions across the entire liver section. White scale bar = 20 µm. **(L)** n=4-7/group. Bars: mean ± SEM. Each dot represents a single biological replicate. P-values were analyzed with two-way ANOVA **(B–G, K)**. ****p ≤ 0.0001,**p ≤ 0.01, *p ≤ 0.05, ns, not significant.

To substantiate the importance of citrin LOF in the development of a disrupted lipid metabolism in TNF-induced SIRS, we measured several lipid metabolic parameters 8h after TNF challenge in Slc25a13^+/+^ and Slc25a13^-/-^ mice (**Figure 4A**). As expected, TNF injection led to significantly more severe hypothermia in Slc25a13^-/-^ mice (**Figure 4B**). Both plasma FFA and glycerol levels were significantly increased in TNF-treated Slc25a13^-/-^ mice compared to PBS controls, a difference that was not (yet) observed in Slc25a13^+/+^ mice (**Figures 4C, D**). This indicates the earlier presence of a dysregulated lipid metabolism in TNF-treated Slc25a13^-/-^ mice. We next determined the potential presence of enhanced lipolysis responsible for the more severe increase in plasma FFA and glycerol levels in TNF-treated Slc25a13^-/-^ mice by determining loss of fat tissue. The total body weight and iWAT weight of both Slc25a13^+/+^ and Slc25a13^-/-^ mice were not altered upon TNF injection (**Figures 4E, F**). Additionally, gene expression of *Lipe* (encodes hormone sensitive lipase enzyme involved in the hydrolysis of triglycerides into FFA and glycerol) in iWAT was even significantly reduced in Slc25a13^+/+^ upon TNF injection compared to PBS controls. This decrease was also observed in both PBS-treated and TNF-treated Slc25a13^-/-^ mice (**Figure 4G**). This suggests no clear detection of enhanced lipolysis in TNF-treated Slc25a13^-/-^ mice, despite other studies showing elevated lipolysis upon TNF treatment (23–25).

Given the more pronounced reduced liver NAD^+^ and NADH levels in TNF-treated Slc25a13^-/-^ mice, and the dependence of mitochondrial FFA β-oxidation on a proper NAD^+^/NADH ratio, we next explored whether citrin LOF contributes to impaired FFA β-oxidation in TNF-induced SIRS. Heatmap depiction of the LFCs of genes involved in FFA β-oxidation showed a more severe downregulation in Slc25a13^-/-^ mice compared to Slc25a13^+/+^ mice 8h post-TNF injection (**Figure 4H**). We assessed mitochondrial FFA β-oxidation capacity via Seahorse analysis on isolated liver mitochondria of PBS- and TNF-treated Slc25a13^+/+^ and Slc25a13^-/-^ mice (**Figure 4I**). Palmitoylcarnitine-driven OCR was already markedly diminished in Slc25a13^-/-^ mitochondria compared to Slc25a13^+/+^ controls under basal conditions (**Figure 4J**, **Supplementary Figure S4A**). Following TNF injection, this reduction became significantly more pronounced in Slc25a13^-/-^ mitochondria, whereas no such decline was observed in Slc25a13^+/+^ mitochondria (**Figure 4J**, **Supplementary Figure S4A**). Hence, citrin deficiency aggravates mitochondrial FFA β-oxidation supposedly leading to the more rapid FFA accumulation in the blood in TNF-induced SIRS. When fatty acid levels are elevated in circulation, the liver stores them in lipid droplets (50). We performed LipidTOX staining to examine the occurrence of ectopic lipid accumulation in livers of PBS-treated and TNF-treated Slc25a13^+/+^ and Slc25a13^-/-^ mice. An increased number of lipid droplets were detected in livers of Slc25a13^-/-^ mice under basal conditions, likely reflecting their diminished mitochondrial FFA β-oxidation capacity (**Figures 4K, L**). Interestingly, liver lipid droplet count was increased in Slc25a13^+/+^ mice upon TNF challenge which was even more augmented in Slc25a13^-/-^ mice (**Figures 4K, L**). Overall, these data illustrate that citrin LOF-mediated MAS dysfunction impairs mitochondrial β-oxidation, ultimately resulting in FFA accumulation and lipid droplet formation in TNF-induced SIRS.

### Citrin knockout exacerbates aerobic glycolysis and lactate clearance leading to lactate-mediated lethal shock in TNF-induced SIRS

**Figure 5:**
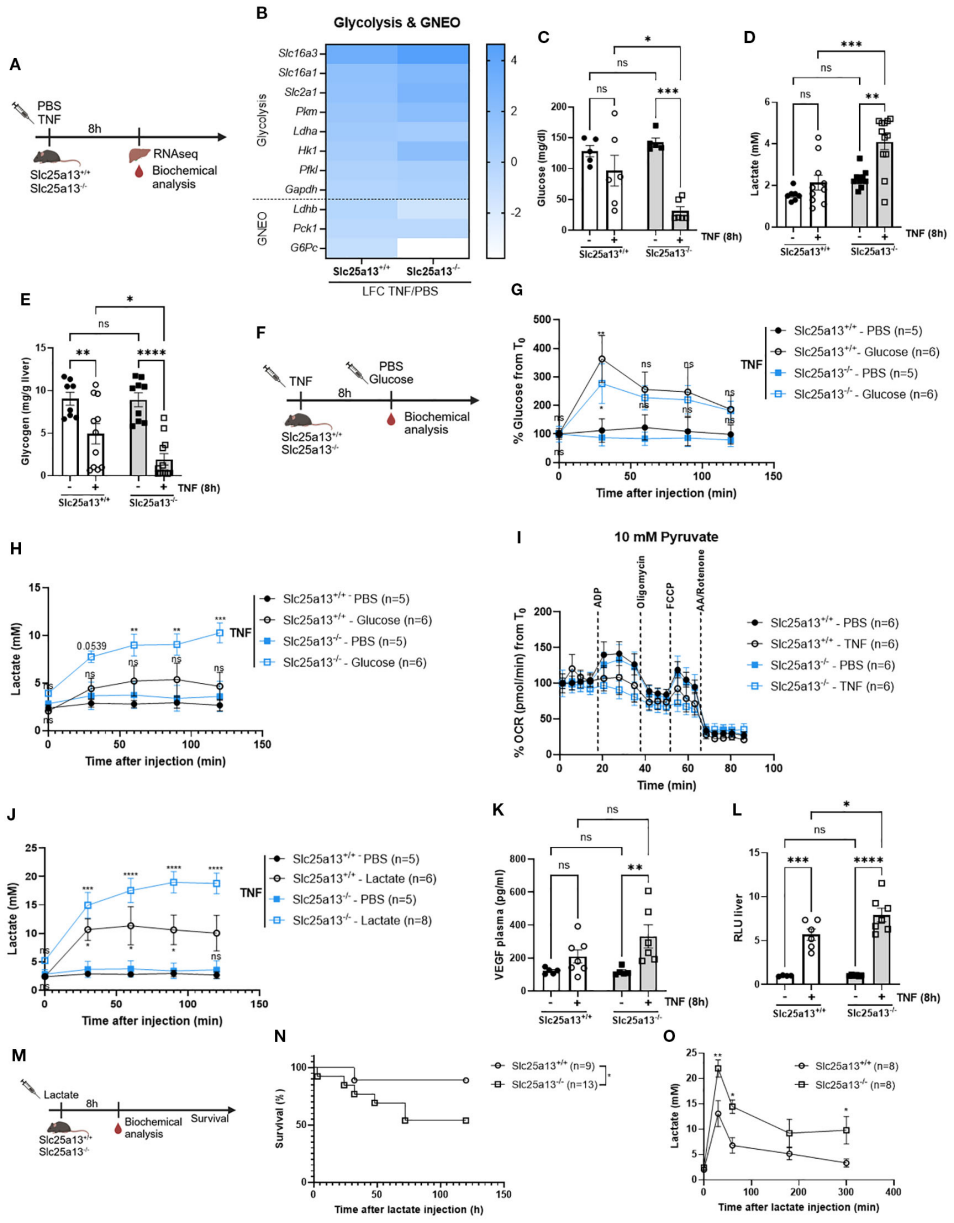
Citrin knockout exacerbates aerobic glycolysis and lactate clearance leading to a lactate-mediated lethal shock in TNF-induced SIRS. **(A)** Experimental setup 8h after TNF (35 µg). **(B)** Heatmap depicting the LFC (TNF versus PBS) of glycolysis and GNEO genes in Slc25a13^+/+^ and Slc25a13^-/-^ mice. **(C–E)** Blood glucose **(C)**, blood lactate **(D)** and liver glycogen **(E)** levels of PBS- and TNF treated Slc25a13^+/+^ and Slc25a13^-/-^ mice. n=5-12/group. **(F)** Experimental setup for glucose tolerance 8h after TNF (35 µg) **(G, H)**. Glucose tolerance in TNF-treated Slc25a13^+/+^ and Slc25a13^-/-^ mice. Blood glucose levels **(G)** and blood lactate levels **(H)** were measured via the tail vein. P-values of glucose injected mice were compared with their respective PBS control. n=5-6/group. **(I)** OCR flux of isolated liver mitochondria of PBS- and TNF-treated Slc25a13^+/+^ and Slc25a13^-/-^ mice, driven by 10 mM pyruvate. n=6/group. **(J)** Lactate tolerance in TNF-treated Slc25a13^+/+^ and Slc25a13^-/-^ mice. Blood lactate levels were measured via the tail vein. P-values of lactate injected mice were compared with their respective PBS control. n=5-8. **(K)** Plasma VEGF levels of PBS- and TNF-treated Slc25a13^+/+^ and Slc25a13^-/-^ mice. n=4-6/group. **(L)**. Vascular permeability in liver of PBS- and TNF-treated Slc25a13^+/+^ and Slc25a13^-/-^ mice. n=4-7/group. **(M)** Experimental setup for lactate survival. **(N, O)** Slc25a13^+/+^ and Slc25a13^-/-^ were IP injected with sodium-L-lactate (3 g/kg) and mortality **(N)** and blood lactate levels **(O)** were monitored. n=8-13/group. Bars: mean ± SEM. Each dot represents a single biological replicate. P-values were analyzed with two-way ANOVA **(C–E, G, H, J–L, O)**. Survival curve was analyzed with a chi-square test **(N)**. ****p ≤ 0.0001, ***p ≤ 0.001, **p ≤ 0.01, *p ≤ 0.05, ns, not significant.

Since TNF-treated Slc25a13^-/-^ mice were characterized by more prominent hyperlactatemia (**Supplementary Figure S3D**) and adequate NAD^+^ and NADH levels are crucial for sustaining glycolysis and oxidative phosphorylation, we next investigated the impact of citrin LOF on carbohydrate metabolism in TNF-induced SIRS. Heatmap visualization of LFCs of genes involved in glycolysis demonstrated a stronger upregulation in Slc25a13^-/-^ mice compared to Slc25a13^+/+^ mice 8h post-TNF injection (**Figures 5A, B**). This was accompanied by more severe hypoglycemia and hyperlactatemia in TNF-treated Slc25a13^-/-^ mice, which was not yet evident in TNF-treated wildtype controls (**Figures 5C, D**) at this 8h post TNF timepoint. Liver glycogen depletion, already observed in TNF-treated Slc25a13^+/+^ mice, was significantly more pronounced in TNF-treated Slc25a13^-/-^ (**Figure 5E**), suggesting accelerated glucose mobilization and glycolytic flux. To further assess glycolytic capacity, a glucose tolerance test was performed upon TNF challenge (**Figure 3F**). Glucose injection resulted in a significant blood glucose increase in TNF-treated Slc25a13^+/+^ which was remarkably less pronounced in Slc25a13^-/-^ mice, indicating quick enhanced glucose utilization (**Figure 5G**). This was accompanied by more severe lactate production in TNF-treated Slc25a13^-/-^ compared to Slc25a13^+/+^ controls (**Figure 5H**), demonstrating the presence of enhanced aerobic glycolysis. Pyruvate-driven OCR was also significantly more diminished in isolated liver mitochondria from Slc25a13^-/-^ mice compared to Slc25a13^+/+^ controls following TNF injection (**Figure 5I**, **Supplementary Figure S4B**). Together, these findings indicate that citrin absence – and the resulting MAS inactivity - contributes to the metabolic switch toward a Warburg-like phenotype in TNF-induced SIRS. Specifically, the accelerated decline in NAD^+^ levels in Slc25a13^-/-^ mice likely drives lactate dehydrogenase A (LDHA)-mediated lactate production to sustain NAD^+^ regeneration for glycolytic flux. Simultaneously, diminished mitochondrial NADH availability may impair pyruvate-driven respiration, further enhancing aerobic glycolysis and excessive lactate production.

In addition to exacerbating lactate production, we propose that citrin LOF also impairs lactate-mediated GNEO in TNF-induced SIRS. Heatmap depiction of genes involved in lactate-mediated GNEO (*Ldhb*, *Pck1* and *G6Pc*) were notably more downregulated in Slc25a13^-/-^ mice compared to Slc25a13^+/+^ mice 8h post-TNF injection (**Figure 5B**). To functionally evaluate lactate clearance, lactate tolerance was assessed in TNF-treated Slc25a13^+/+^ and Slc25a13^-/-^ mice. Lactate administration caused a significant increase in blood lactate in the TNF-treated Slc25a13^+/+^ mice. This increase was remarkably more apparent in TNF-treated Slc25a13^-/-^ mice (**Figure 5J**). Hence, it is suggested that MAS dysfunction due to citrin absence leads to diminished lactate clearance in TNF-induced SIRS by a cytosolic NAD^+^ shortage as the LDHB-catalyzed conversion of pyruvate to lactate is NAD^+^ dependent.

Interestingly, a previous study showed that elevated blood lactate levels, in the absence of efficient clearance, can induce toxicity through lactate-mediated VEGF production, promoting vascular permeability and ultimately resulting in lethal vascular collapse (51). Based on these findings, we hypothesized that the heightened sensitivity to TNF-induced SIRS observed in Slc25a13^-/-^ mice is driven by lactate-mediated toxicity. Indeed, TNF treatment resulted in significantly higher VEGF levels (**Figure 5K**) and a stronger upregulation of genes related to endothelial activation and damage in Slc25a13^-/-^ mice compared to wildtype controls (**Supplementary Figure S4C**). This was associated with more vascular permeability for most organs (**Figure 5L**, **Supplementary Figures S4D–G**). To corroborate these findings, lactate-induced toxicity was assessed in Slc25a13^+/+^ and Slc25a13^-/-^ mice under basal conditions (**Figure 5M**). Slc25a13^-/-^ mice displayed significantly higher mortality for a sublethal lactate dose compared to wildtype controls (**Figure 5N**). This was correlated with diminished lactate clearance, reflected by significantly elevated blood lactate levels over time in Slc25a13^-/-^ mice compared to wildtype controls (**Figure 5O**). Overall, this shows that citrin deficiency-induced hyperlactatemia is a key driver for lactate-mediated VEGF production and vascular permeability, thereby contributing to lethality in TNF-induced SIRS.

## Discussion

Significant metabolic alterations in carbohydrate and fat metabolism are key hallmarks of SIRS, as extensively evidenced in other studies (18, 52–54). We substantiate that TNF-induced SIRS is characterized by (1) a metabolic switch from oxidative phosphorylation towards aerobic glycolysis (i.e. Warburg-like phenotype), (2) reduced lactate-mediated GNEO, and (3) diminished mitochondrial FFA β-oxidation. These phenomena lead to severe hypoglycemia, liver glycogen depletion, hyperlactatemia and elevated blood FFA levels, all contributing to TNF-induced lethal shock. However, the potential mechanisms behind these metabolic deviations are still a matter of debate. According to the literature, TNF-induced Warburg-like metabolism can be the result of NF-κB or iNOS dependent HIF1α activation (15–17, 20), Akt-mTOR signaling by the kinase ITK (55) or excessive ROS production and oxidative stress resulting in mitochondrial dysfunction (56, 57). Also, impaired PPARα activity (58) or PGC1a dysfunction (28–30) leading to the downregulation of β-oxidation enzymes are possible explanations for disruptive mitochondrial FFA β-oxidation in SIRS. However, a coherent mechanism that explains both the presence of the Warburg phenotype and reduced FFA β-oxidation, *in vivo* during SIRS, still remains elusive. The acute induction of SIRS by TNF in mice is an ideal model to dissect the underlaying mechanisms.

One of the main features of both glycolysis and FFA β-oxidation is their reliance on a continuous regeneration of cytosolic NAD^+^ and generation of mitochondrial NADH, mediated by two redox shuttles, i.e. the MAS and, to a lesser extent, the glycerol-3-phosphate shuttle. In this study, we found that TNF impairs MAS activity leading to a NAD^+^ and NADH shortage. Our findings align with previous studies illustrating the presence of disruptive ionic shuttles in hemorrhagic shock and TNF-mediated MAS disruption and mitochondrial dysfunction in enteric neurons derived from patients with Parkinson’s disease (59, 60). We propose that TNF-induced MAS inactivity is mediated by *Slc25a13* downregulation due to HNF4α LOF. TNF is known to inhibit HNF4α transcriptional activity, potentially through NF-κB activation which may impair HNF4α DNA binding affinity and transactivation potential (45, 61, 62). Other possibilities include FOXA2 and USF1, two transcription factors that are known to regulate *Slc25a13* expression, and FOXA2 has been shown to be downregulated during systemic inflammation (63, 64).

Of note, some studies proposed that TNF and other inflammatory stimuli also affect NAD^+^ biosynthesis. For instance, TNF treatment upregulates the expression of both major NAD^+^-consuming enzymes (e.g. CD38) and NAD^+^-synthesizing enzymes (e.g. IDO), ultimately resulting in diminished cellular NAD^+^ levels in primary macrophages (65–67). In contrast, LPS induced TNF release has also been shown to correlate with increased NAD^+^ levels in pro-inflammatory macrophages, highlighting a potential interplay between NAD^+^ and inflammatory responses (68). Moreover, research demonstrated that the presence of Warburg metabolism in LPS-stimulated pro-inflammatory macrophages relies on NAD^+^ metabolism. This is mediated by mitochondrial ROS causing oxidative DNA damage leading to NAD^+^ depletion and upregulation of NAD^+^ salvage pathways, thereby restoring NAD^+^ levels and sustaining the Warburg phenotype and inflammation (69–72).

By using Slc25a13^-/-^ mice to eliminate MAS activity, we demonstrate that impaired redox shuttling underlies the prominent lipid metabolic disturbances in TNF-induced SIRS. Our data reveal that TNF’s disruptive hepatic mitochondrial FFA β-oxidation is aggravated in Slc25a13^-/-^ mice, leading to accelerated circulatory FFA accumulation and hepatic steatosis. In our opinion, there are two possible explanations for this observation. First, because NAD^+^ deficiency has been shown to promote hepatic steatosis in a diet-induced Metabolic dysfunction-associated steatotic liver disease model (73). TNF-induced MAS dysfunction in combination with impaired oxidative phosphorylation capacity, may indirectly reduce mitochondrial NAD^+^ levels. Because one of the rate-limiting enzymes of FFA oxidation (3-hydroxyacyl-CoA dehydrogenase) requires NAD^+^ as a cofactor, NAD^+^ depletion would hinder its activity and lead to reduced FFA oxidation and to lipid accumulation (74–76). Second, transcriptional repression of genes encoding mitochondrial FFA B-oxidation enzymes due to PPARα inactivity. Dyslipidemia and steatogenesis in patients with citrin deficiency have also been associated with PPARα downregulation, but the mechanisms behind this downregulation remained elusive (77). Interestingly, LOF of the NAD^+^-dependent deacetylase SIRT1 has been associated with impaired FFA oxidation and hepatic steatosis in fasted mice and during a high-fat diet. This impairment is mediated through the absence of SIRT1-mediated deacetylation and activation of PGC1α (78, 79). PGC1a is an important coactivator of many nuclear receptors that regulate cellular energy metabolic pathways and its interaction is crucial for constitutive activity of PPARα (80). We propose that NAD^+^ depletion due to TNF-induced MAS inactivity hinders SIRT1-mediated deacetylation of PGC1α, eventually resulting in PPARα inactivity and massive transcriptional repression. Interestingly, the administration of resveratrol, a SIRT1 activator, has been reported to ameliorate mitochondrial function, aerobic capacity and FFA oxidation by activating SIRT1 and PGC1α (81, 82). Hence, this warrants further exploration.

Furthermore, we found that TNF-induced MAS dysfunction plays a crucial role in driving the metabolic switch towards a Warburg-like metabolism in SIRS. This was characterized by accelerated glucose utilization, exacerbated lactate production, and further impairment of mitochondrial pyruvate-driven respiration in TNF-treated Slc25a13^-/-^ mice. However, patients with citrin deficiency display impaired hepatic glycolysis as cytosolic NAD^+^ levels are heavily depleted due to MAS inactivity (41, 43), which is in strong contrast with our observations. Yet, other studies suggest that the lack of cytosolic NAD^+^ regeneration in MAS-deficient cells can be compensated by the activation of other NAD^+^-recycling pathways, like the glycerol-3-phosphate shuttle and LDHA (48, 49, 83). While the glycerol-3-phosphate shuttle provides some backup NAD^+^ recycling capacity, it is less active and less efficient compared to the MAS and its maximal activity is rapidly reached (84–86). Furthermore, when the glycolytic rate exceeds the NAD^+^-regenerating capacity of both mitochondrial NADH shuttles, this is accompanied by increased lactate excretion (87, 88). Pyruvate reduction via elevated LDHA activity then becomes the primary route for NAD^+^ regeneration. Given that TNF is a well-known glycolytic inducer, we propose that in combination with TNF-induced MAS inactivity, TNF-activated glycolysis will be further accelerated by (1) enhanced LDHA activity responsible for NAD^+^ regeneration, and (2) by diminished mitochondrial pyruvate-driven respiration due to restricted mitochondrial NADH availability, all leading to excessive lactate production in TNF-induced SIRS.

Prominent hyperlactatemia in TNF-treated Slc25a13^-/-^ mice not only results from enhanced production via LDHA activity for NAD^+^ regeneration, but also from more increased lactate clearance problems (via GNEO). Impaired GNEO starting from lactate is a well-known phenomenon in citrin deficient individuals, and is thought to result from the altered cytosolic NADH and NAD^+^ levels (43, 48). Deficiency in nicotinamide riboside kinase 1, a rate-limiting enzyme in NAD^+^ synthesis, has indeed been shown to diminish the gluconeogenic potential (89). Hence, in our context, it seems logical that the available NAD^+^ is scavenged by the glycolysis pathway, hindering the essential NAD^+^-dependent conversion of lactate to pyruvate via LDHB during lactate-mediated GNEO. It is also possible that TNF-induced MAS inactivity induces a shift in the LDH isozyme profile, leading to lower LDH1 and LDH2 levels and increased LDH4 and LDH5 levels, as was observed during hypoxic conditions, to promote NAD^+^ recycling (90).

We provide evidence that elevated blood lactate levels, due to MAS inactivity contribute to lethality in TNF-induced SIRS by promoting VEGF-mediated vascular leakage, a mechanism that has been unfolded in sepsis (51). Restoring NAD^+^ deficits or NAD^+^ recycling, could therefore be an interesting therapeutic target to limit hyperlactatemia and it’s toxic effects. Supplementation with the NAD^+^ precursor, nicotinamide riboside, could be valuable as it has been shown to improve the dysregulated carbohydrate metabolism and FFA-β-oxidation in citrin deficient hepatocytes (91). It also attenuated ethanol-induced inflammation by activating SIRT1, thereby limiting metabolic changes in the glycolytic and oxidative phosphorylation pathways, and mitigating lactate release in macrophages (92).

In summary, our data demonstrate that TNF-induced SIRS is marked by MAS inactivity, possibly by a transcriptional downregulation of the *Slc25a13* gene due to TNF-induced HNF4α inactivation. The MAS problem is leading to significant metabolic dysregulations. Defective cytosolic NAD^+^ recycling as a result of MAS dysfunction causes a shift towards enhanced lactate production via LDHA and diminished lactate clearance via LDHB in an attempt to ensure NAD^+^ regeneration for TNF-induced glycolysis. This imbalance contributes to severe hypoglycemia, hyperlactatemia and lethality. Additionally, NAD^+^ deficits play a crucial role in diminished mitochondrial FFA-β-oxidation resulting in circulatory FFA accumulation and hepatic steatosis.

## Materials and methods

### Mice

C57BL6/J (wildtype) mice were purchased from Janvier (Le Genest-St. Isle, France). Mutant Hnf4a^fl/fl^ mice were generated by Dr. Frank Gonzalez (NIH, Bethesda, USA) and formally called B6.129×1(FVB)-Hnf4atm1.1Gonz/J (93). The mice had been backcrossed into C57BL/6J background and were provided by Dr. Iannis Talianidis (University of Crete, Heraklion (Greece), by courtesy of Dr. Frank Gonzalez, and were under protection of an MTA. AlbCreERT2^Tg/+^ mice were kindly provided by Dr. D. Metzger & Dr. P. Chambon (Igbmc, France) (94). Hnf4α^fl/fl^;AlbCreERT2^+/+^ (Hnf4α^fl/fl^) and Hnf4α^fl/fl^;AlbCreERT2^Tg/+^ (Hnf4α^Liver-i-KO^) were generated by crossing as described by Van Dender et al. (44). Hnf4α^fl/fl^ and Hnf4α^Liver-i-KO^ were intraperitoneally (IP) injected with 1 mg tamoxifen in a 1:8 ethanol:oil solution for 5 consecutive days to induce hepatic HNF4α depletion, which was observed 3 days after the final tamoxifen injection. Slc25a13^-/-^ mice, in a C57BL6/J background, were generated in house by the transgenic core facility (IRC-VIB, Ghent University, Belgium) and formally called Slc25a13^em1Clib^. A deletion of 83 base pairs was induced in exon 6 via Crispr/Cas. Slc25a13^-/-^ mice were backcrossed to C57BL/6J (wildtype) mice, purchased from Janvier (Le Genest-St. Isle, France), to establish Slc25a13^-/-^ and Slc25a13^+/+^ mice by Slc25a13^+/-^ intercrosses. All mice were housed in individually ventilated cages at conventional housing conditions (22°C, 14/10h light/dark cycle, dark phase starting at 9 p.m.) in a specific pathogen-free facility. Food (chow diet consisting of 18% proteins, 4.5% fibers, 4.5% fat and 6.3% ashes, Provimi Kliba SA) and water were given *at libitum*, unless if otherwise stated. Male and female mice were used at the age of 8–20 weeks. Approval for all experiments was granted by the Institutional Ethics Committee for animal welfare of the Faculty of Sciences, Ghent University, Belgium. The methods were in conformity with the relevant guidelines and regulations.

### Injections and sampling

All injections were administered IP and injection volumes were adapted based on the bodyweight of the mice. Mice were injected with recombinant mouse TNF in a volume of 200 µL/20g. A dose of LD_50_ or LD_100_ of TNF was determined in advance and varies depending on the mouse strain, animal house, type of experiment and the TNF batch. Doses are depicted in the figure legends. Recombinant mouse TNF was generated in *Escherichia coli* and purified at our facility in the absence of detectable endotoxin contamination.

During lethality experiments, the rectal body temperature of the mice was frequently monitored, and when it dropped below 28°C, the mice were euthanized via cervical dislocation (humane endpoint). For sampling experiments, blood (for plasma isolation) was collected via cardiac puncture after anesthetizing the mice with ketamine (100 mg/kg) and xylazine (10 mg/kg) mixture. Mice were euthanized via cervical dislocation at the indicated timepoints and organs were isolated. Organs were preserved in RNA later (Life Technologies Europe) or snap-frozen in liquid nitrogen for further analysis.

To assess glucose and lactate tolerance, glucose monohydrate (2 g/kg; Sigma 49159) or sodium-L-lactate (2 g/kg; Sigma 71718) were both dissolved in PBS and administered 8h post TNF injection. To determine lactate toxicity, mice were injected with sodium-L-Lactate (≥ 3 g/kg; dissolved in PBS; Sigma 49159) and lethality was monitored.

### Biochemical analysis of fluids or tissues

Blood lactate and glucose levels were monitored in tail blood using the Lactate Plus meter (NOVA Biomedical) and the OneTouch Verio glucose meter, respectively. Plasma FFA (Abnova KA1667), plasma glycerol (Cayman Chemical 10010755-96), liver glycogen (Abcam ab65620) and liver NAD^+^ and NADH (Abcam ab65348) levels were measured via a colorimetric assay kit. Plasma VEGF levels (Bio-Techne MMV00) were quantified via ELISA. All assays were executed according to the manufacturer’s protocol.

### Western blot analysis

For the detection of SLC25A13 and SLC25A11, total protein was isolated from snap-frozen liver tissue with RIPA lysis buffer containing a protease inhibitor cocktail (Roche). Protein concentration was determined by Bradford assay (Bio-Rad). Protein samples (50 µg protein) mixed with loading dye were separated by electrophoresis on a 8% gradient SDS-polyacrylamide gel, followed by transfer onto a nitrocellulose membrane (pore size 0.45 µm). The membranes were blocked with a ½ dilution of Starting Block/PBST0.1% (Thermo Fisher Scientific) followed by an overnight incubation at 4 °C with primary antibodies against SLC25A13 (1:1000; NBP1-33380, Novus Biologicals) or SLC25A11 (1:1000; PA5-27510; Thermofisher Scientific), and β-ACTIN (1:5000; MA5-15739, Thermo Fisher Scientific) or β-TUBULIN (1:1000; 2146S, Cell Signaling Technology) as loading control. After washing with PBST0.1%, the blots were incubated with Amersham ECL anti-mouse antibody (1:2000, GENA931, GE Healthcare Life Sciences) or Amersham ECL anti-rabbit antibody (1:2000; GEN934, GE Healthcare Life Sciences) for 1h at room temperature. The blots were washed with PBST0.1% and immunoreactive bands were detected and quantified using the WesternBright ECL kit (Advansta Inc.) and an Amersham Imager 600 (GE Healthcare Life Sciences).

### Transcriptomics analysis

#### RNA sequencing

##### Liver – TNF 18h dataset

We used the liver TNF dataset GSE237949 that was processed as described by Wallaeys et al. (46). Gene level read counts were obtained using feature Counts and differentially expressed genes were identified with the DESeq2 R package, setting the false discovery rate (FDR) at 5% (95, 96).

##### Liver – Hnf4a^Liver-i-KO^ dataset

We used the liver Hnf4a^Liver-i-KO^ dataset GSE260635 that was processed as described by Van Dender et al. (44). Differentially expressed genes were identified using the DESeq2 R package with a FDR at 5% (95, 96).

##### Liver – Slc25a13^-/-^ TNF 8h dataset

Total RNA was isolated with Aurum Total RNA mini Kit (Bio-Rad) following the manufacturer’s instructions. RNA concentration and quality were determined with the Agilent RNA 6000 Pico Kit (Agilent Technologies). RNA was used to construct an Illumina sequencing library using the Illumina TruSeqLT stranded RNA-seq library protocol, and single-end sequencing was performed on an element AVITI instrument (VIB Nucleomics Core). The obtained reads were mapped to the mouse (mm39) reference genome using STAR v2.7.10b (97). Multimapping reads were removed. Gene level read counts were directly aquired from STAR using the --quantMode GeneCounts flag and differentially expressed genes were acquired using DESeq2 package, with the FDR set at 5% (95, 96).

Enrichr, Metascape and HOMER were used for further RNASeq data analysis (98–100).

### Real-time qPCR

White adipose tissue was isolated, snap-frozen and stored at -20°C. The Aurum Total RNA mini Kit (Bio-Rad) was used for total RNA isolation following the manufacturer’s instructions. RNA concentration and quality were measured using the Nanodrop 1000 (Thermo Scientific) and 1000 ng RNA was used for cDNA synthesis with the Sensifast cDNA Synthesis Kit (Bioline). cDNA was 10 time diluted in nuclease-free water. RT-qPCR was executed using the Bioline SensiFAST SYBR No-ROX mix (Bioline) and the Roche LightCycler 480 system (Applied Biosystems). Genorm was used to determine the stability of the housekeeping genes and qPCR data were analyzed with qBase+ software (Biogazelle). Results are depicted as relative expression values normalized to the geometric mean of the housekeeping genes. Used qPCR primers are depicted in **Table 1**.

**Table 1:** List of primer sequences used for RT-qPCR.

Gene	Forward primer (5’-3’)	Reverse primer (5’-3’)
*Gapdh*	TGAAGCAGGCATCTGAGGG	CGAAGGTGGAAGAGTGGGAG
*Lipe*	CCAGCCTGAGGGCTTACTG	CTCCATTGACTGTGACATCTCG
*Tbp*	GAAGCTGCGGTACAATTCCAG	CCCCTTGTACCCTTCACCAAT

### Liver mitochondria isolation

Liver mitochondria were isolated following the protocol in Frezza et al. (101). In short, the whole liver was isolated, washed and minced in ice-cold isolation buffer (1 M sucrose, 0.1 M Tris/MOPS and 0.1 M EGTA/Tris). A Dounce tissue grinder set (Sigma D9063) was used to homogenize the minced liver. After a centrifugation step (600 g, 10min, 4°C), the supernatans was collected and recentrifuged at 7000 g, 10 min, 4°C. The resuspended pellet in ice-cold isolation buffer was recentrifuged at 7000 g, 10 min, 4 °C to collect purified mitochondria. The total mitochondrial protein content was measured via a Bradford protein assay (Bio-Rad).

### Seahorse analysis

The OCR of isolated liver mitochondria was assessed using a Seahorse Bioscience XF96 Analyzer (Agilent), following the protocol by Luso et al. (102). Mitochondria (10 µg) in mitochondrial assay solution (MAS, 70 mM sucrose, 220 mM mannitol, 10 mM KH_2_PO_4_, 5mM MgCl_2_, 2 mM HEPES, 1 mM EGTA and 0.2% fatty acid-free BSA) were delivered/well in a 96-well Seahorse microplate (Agilent). After centrifugation (20 min, 2000 g, 4°C), prewarmed MAS with specific respiratory substrates (40 µM palmitoylcarnitine and 0.5 mM malate or 10 mM pyruvate) were administered to the mitochondria, followed by a 10 min incubation at 37°C. The OCR flux was determined over time after the sequential injection of ADP (40 mM), oligomycin (25 µg/ml), FCCP (40 µM) and rotenone (20 µM) with antimycin A (AA; 40 µM).

### LipidTOX

Isolated liver tissue was fixed in antigenfix (DiaPath) at 4 °C for 1-2h. Liver tissues were washed with PBS, followed by an overnight incubation in 34% sucrose at 4 °C, and were mounted with O.C.T. compound (Tissue-Tek). Cryostat sections of 20 µm thickness were rehydrated in PBS and were blocked for 30 min at room temperature (RT) with blocking buffer (2% BSA, 1% fetal calf serum, 1% goat serum, in 0.5% saponin). The liver sections were incubated with a primary antibody mix (LipidTOX Deep Red (1:400; Life Technologies Europe B.V.); Acti-stain 488 Phalloidin (1:150; Cytoskeleton Inc.)) for 2h at RT. The slides were washed with PBS and the nuclear staining (DAPI (1:1000, Sigma-Aldrich N.V.)) was added for 15 min at RT. After washing with PBS and bidi to remove residual salts, the slides were mounted with PVA including DABCO. For each liver section, Z-stacks of 8 areas were imaged using a spinning disk confocal microscope (Zeiss), with a Plan-Apochromat 40x/1.4 oil DIC (UV) VIS-IR M27 objective lens at a pixel size of 0.275 µm and at optimal Z-resolution (240 mm). Z-stacks were analyzed in Arivis and the amount of lipid droplets relative to the tissue volume was quantified.

### Vascular permeability assay

8h after TNF injection, mice were intravenously injected with FITC-dextran 4 kDa (25 mg/kg, TDB labs 60842-46-8). One hour post-injection, mice were transcardially perfused with 0.2% EDTA in PBS after anesthetizing the mice with ketamine (100 mg/kg) and xylazine (10 mg/kg) mixture. Organs were isolated, cut in small pieces and overnight incubated (37°C; shaking) in 100% formamide (Sigma) for FITC-labeled dextran extraction. After centrifugation (15 min, 14–000 rpm), the supernatans was 1/20 diluted in PBS and fluorescence was measured with a FLUOstar OMEGA plate reader (BMG Labtech, Germany). Fluorescence was normalized to the respective Slc25a13^+/+^ - PBS control per tissue.

### Statistics

Statistical and graphical data analysis were performed using GraphPad Prism software (version 10.3.1). qPCR data was log-transformed to obtain normal distribution. An unpaired Student’s t-test was performed to compare two group means. A two-way ANOVA with a Tukey’s multiple comparisons test was used for experimental setups with a second variable. Kaplan Meier survival curves were compared using a Log-Rank (Mantel-Cox) test or one-side chi-squared test.

Statistical significance was defined by a P-value of < 0.05. **** P < 0.0001, *** P < 0.001, ** P < 0.01, * P < 0.05, ns: not significant. All data are expressed as means ± standard error of the means (SEM). N represents the number of biological replicates used in the experiments. Group sizes were chosen based on prior experience. Statistical details can be found in the figure legends.

## Data Availability

RNASeq data have been deposited at the National Center for Biotechnology Gene Expression Omnibus (GEO) public database and are publicy available as of the date of publication under the accession number GSE309252. This paper also makes use of publicy available datasets, including: RNASeq data from liver 18h after TNF (GEO: GSE237949); RNASeq data from liver HNF4aKO (GEO: GSE260635) and HNF4a CHIPSeq data (GEO: GSE245682).

## Supplementary material

Supplementary Figure 1 TNF-induced SIRS is characterized by severe metabolic reprogramming. **(A–D)**. Enrichr pathway analysis (TRRUST Transcription Factors 2019, MSigDB Hallmark 2020 and GO Biological Process 2023) **(A–C)** and Metascape pathway analysis **(D)** of the upregulated genes 18h after TNF (1502 genes) with LFC > 1 and p < 0.05. **(E)**. % decrease in body weight of PBS- and TNF treated mice, with body weight before injection set at 100%. n=3-5/group. **(F)**. Weight of the inguinal fat pad (iWAT) relative to the total body weight of PBS- and TNF-treated mice (18h). n=3-5/group. **(G, H)**. Calculated basal respiration, ATP-linked respiration and maximal respiration of isolated liver mitochondria of PBS- and TNF treated mice, driven by specific respiratory substrates, i.e. 40 µM palmitoylcarnitine and 0.5 mM malate **(G)** or 10 mM pyruvate **(H)**. n=4-7/group. Bars: mean ± SEM. Each dot represents a single biological replicate. P-values were analyzed with two-way ANOVA **(E, F)** and with unpaired t-test **(G, H)**. *p ≤ 0.05, ns: not significant.

Supplementary Figure 2 TNF impairs the malate aspartate shuttle leading to a NAD^+^ and NADH deficit. **(A)**. mRNA counts of *Slc25a11* in livers of PBS- and TNF-treated mice. n=3/group. **(B, C).** Western blot analysis of SLC25A11 (34 kDa) protein levels in livers of PBS- and TNF-treated mice, normalized to β-tubulin levels (55 kDa). n=3-5/group. **(D).** HNF4α ChIP-Seq analysis of the *Slc25a13* promoter region visualized via Integrated Genome Browser in wildtype mice. n=4/group. **(E).** mRNA counts of *Slc25a13* in livers of Hnf4a^fl/fl^ and Hnf4a^Liver-i-KO^ mice. n=3/group. Bars: mean ± SEM. Each dot represents a single biological replicate. P-values were analyzed with unpaired t-test **(A, B, E)**. **p ≤ 0.01, *p ≤ 0.05, ns: not significant.

Supplementary Figure 3 Citrin knockout results in acute lethality and enhanced metabolic dysfunctions in TNF-induced SIRS. **(A, B)**. Western blot analysis of SLC25A13 (74 kDa) protein levels in livers of Slc25a13^+/+^ and Slc25a13^-/-^ mice, normalized to actin levels (42 kDa). n=3-4/group. **(C, D)**. Body temperature **(C)** and blood lactate levels **(D)** in PBS- and TNF-treated Slc25a13^+/+^ and Slc25a13^-/-^ mice 0h, 4h, 6h and 8h post-injection. P-values of TNF injected mice were compared with their respective PBS control n=5-7/group. **(E)**. Enrichr pathway analysis (MSigDB Hallmark 2020) of the unique upregulated genes (1227) 8h after TNF in Slc25a13^-/-^ mice with LFC > 1 and p < 0.05. **(F)**. Heatmap depicting the LFC (TNF versus PBS) of inflammatory and endothelial activation genes in Slc25a13^+/+^ and Slc25a13^-/-^ mice. **(G)**. Enrichr pathway analysis (ChEA 2022) of the unique downregulated genes 8h after TNF in Slc25a13^-/-^ mice (2164 genes) with LFC < -1 and p < 0.05. **(H)**. Scatter plot depicting the LFCs of differentially expressed genes in 18h TNF-treated Slc25a13^+/+^ mice compared to 8h TNF-treated Slc25a13^-/-^ mice. The black line represents the diagonal (r=1) and the red line indicates the real slope (r= 0.7814) of the data. Data were analyzed with linear regression. Bars: mean ± SEM. Each dot represents a single biological replicate. P-values were analyzed with unpaired t-test **(B)** or with two-way ANOVA **(C, D)**. ***p ≤ 0.001, **p ≤ 0.01, *p ≤ 0.05.

Supplementary Figure 4 Supplementary statistical analysis of seahorse data and vascular permeability. **(A, B)**. Calculated basal respiration, ATP-linked respiration and maximal respiration of isolated liver mitochondria of PBS- and TNF-treated Slc25a13^+/+^ and Slc25a13^-/-^ mice, driven by specific respiratory substrates, i.e. 40 µM palmitoylcarnitine and 0.5 mM malate **(A)** or 10 mM pyruvate **(B)**. n=6/group. **(C)**. Heatmap depicting the LFC (TNF versus PBS) of endothelial activation and damage genes in Slc25a13^+/+^ and Slc25a13^-/-^ mice. **(D–G)**. Vascular permeability in heart **(D)**, kidney **(E)**, lung **(F)** and spleen **(G**) of PBS- and TNF-treated Slc25a13^+/+^ and Slc25a13^-/-^ mice. n=4-7/group. Bars: mean ± SEM. Each dot represents a single biological replicate. P-values were analyzed with two-way ANOVA **(A, B, D–G)**. ****p ≤ 0.0001, ***p ≤ 0.001, **p ≤ 0.01, *p ≤ 0.05, ns: not significant.
