# Supplementary material for: A crucial role of the malate aspartate shuttle in metabolic reprogramming in TNF-induced SIRS
Source: Front Immunol. 2025 Oct 8;16:1652516. doi: 10.3389/fimmu.2025.1652516 (PMC12542566; doi:10.3389/fimmu.2025.1652516)
Supplement: Supplementary Figure 1 — TNF-induced SIRS is characterized by severe metabolic reprogramming. (A–D). Enrichr pathway analysis (TRRUST Transcription Factors 2019, MSigDB Hallmark 2020 and GO Biological Process 2023) (A–C) and Metascape pathway analysis (D) of the upregulated genes 18h after TNF (1502 genes) with LFC > 1 and p < 0.05. (E). % decrease in body weight of PBS- and TNF treated mice, with body weight before injection set at 100%. n=3-5/group. (F). Weight of the inguinal fat pad (iWAT) relative to the total body weight of PBS- and TNF-treated mice (18h). n=3-5/group. (G, H). Calculated basal respiration, ATP-linked respiration and maximal respiration of isolated liver mitochondria of PBS- and TNF treated mice, driven by specific respiratory substrates, i.e. 40 µM palmitoylcarnitine and 0.5 mM malate (G) or 10 mM pyruvate (H). n=4-7/group. Bars: mean ± SEM. Each dot represents a single biological replicate. P-values were analyzed with two-way ANOVA (E, F) and with unpaired t-test (G, H). *p ≤ 0.05, ns: not significant. [file Presentation1.pptx]

## Slide 1
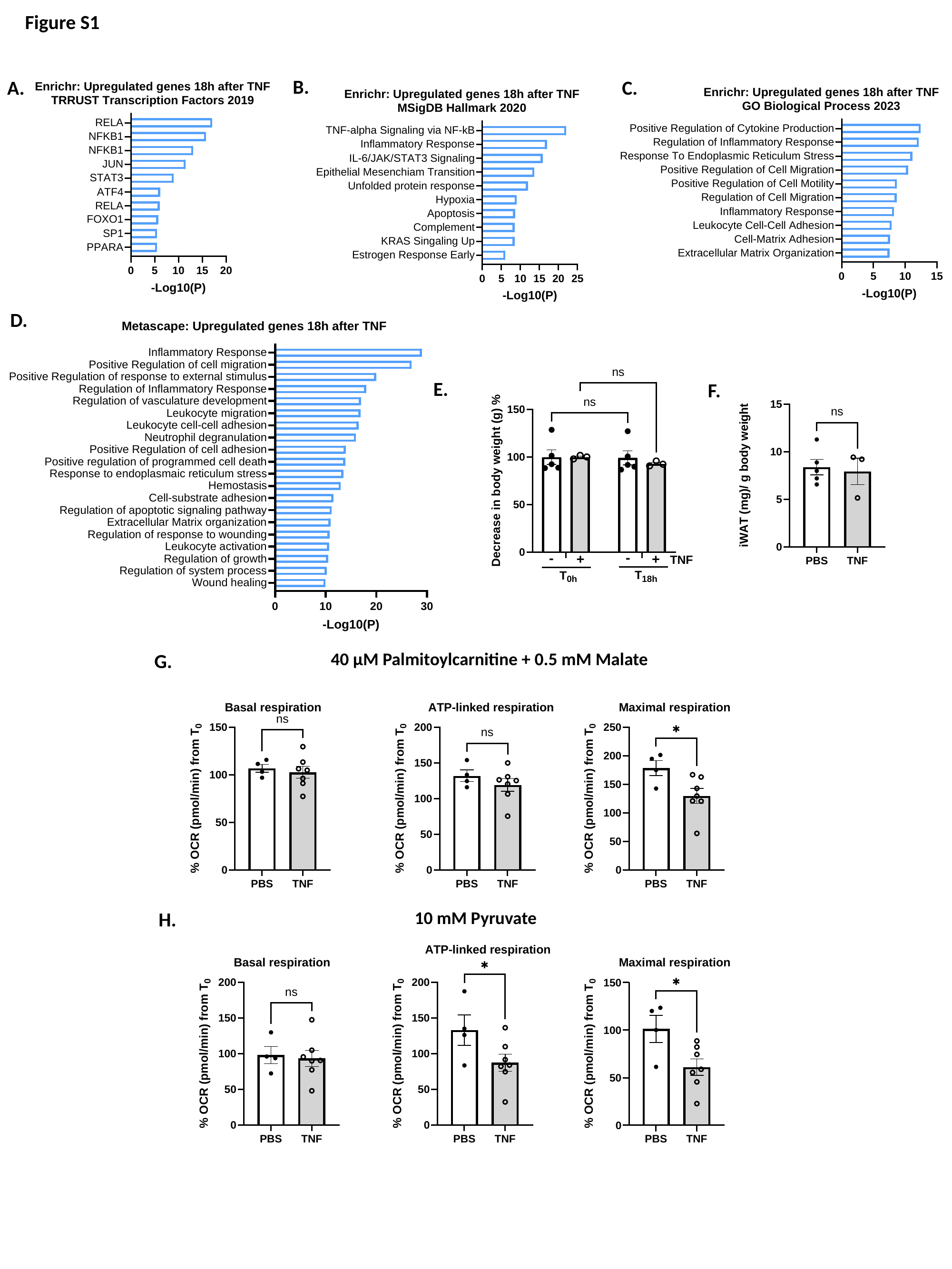

Figure S1
B.
A.
C.
D.
E.
F.
40 µM Palmitoylcarnitine + 0.5 mM Malate
G.
10 mM Pyruvate
H.

## Slide 2
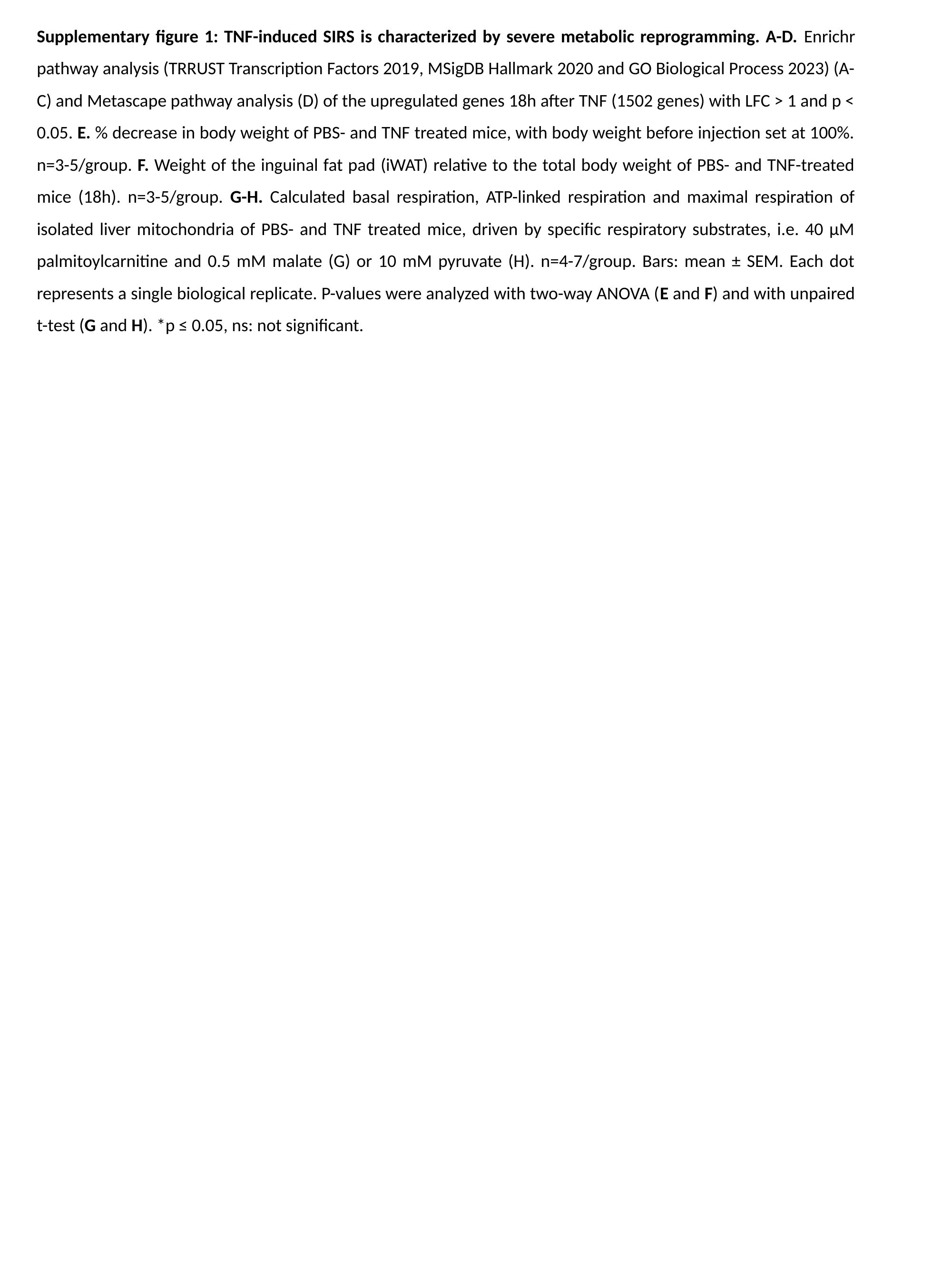

Supplementary figure 1: TNF-induced SIRS is characterized by severe metabolic reprogramming. A-D. Enrichr pathway analysis (TRRUST Transcription Factors 2019, MSigDB Hallmark 2020 and GO Biological Process 2023) (A-C) and Metascape pathway analysis (D) of the upregulated genes 18h after TNF (1502 genes) with LFC > 1 and p < 0.05. E. % decrease in body weight of PBS- and TNF treated mice, with body weight before injection set at 100%. n=3-5/group. F. Weight of the inguinal fat pad (iWAT) relative to the total body weight of PBS- and TNF-treated mice (18h). n=3-5/group. G-H. Calculated basal respiration, ATP-linked respiration and maximal respiration of isolated liver mitochondria of PBS- and TNF treated mice, driven by specific respiratory substrates, i.e. 40 µM palmitoylcarnitine and 0.5 mM malate (G) or 10 mM pyruvate (H). n=4-7/group. Bars: mean ± SEM. Each dot represents a single biological replicate. P-values were analyzed with two-way ANOVA (E and F) and with unpaired t-test (G and H). *p ≤ 0.05, ns: not significant.

## Slide 3
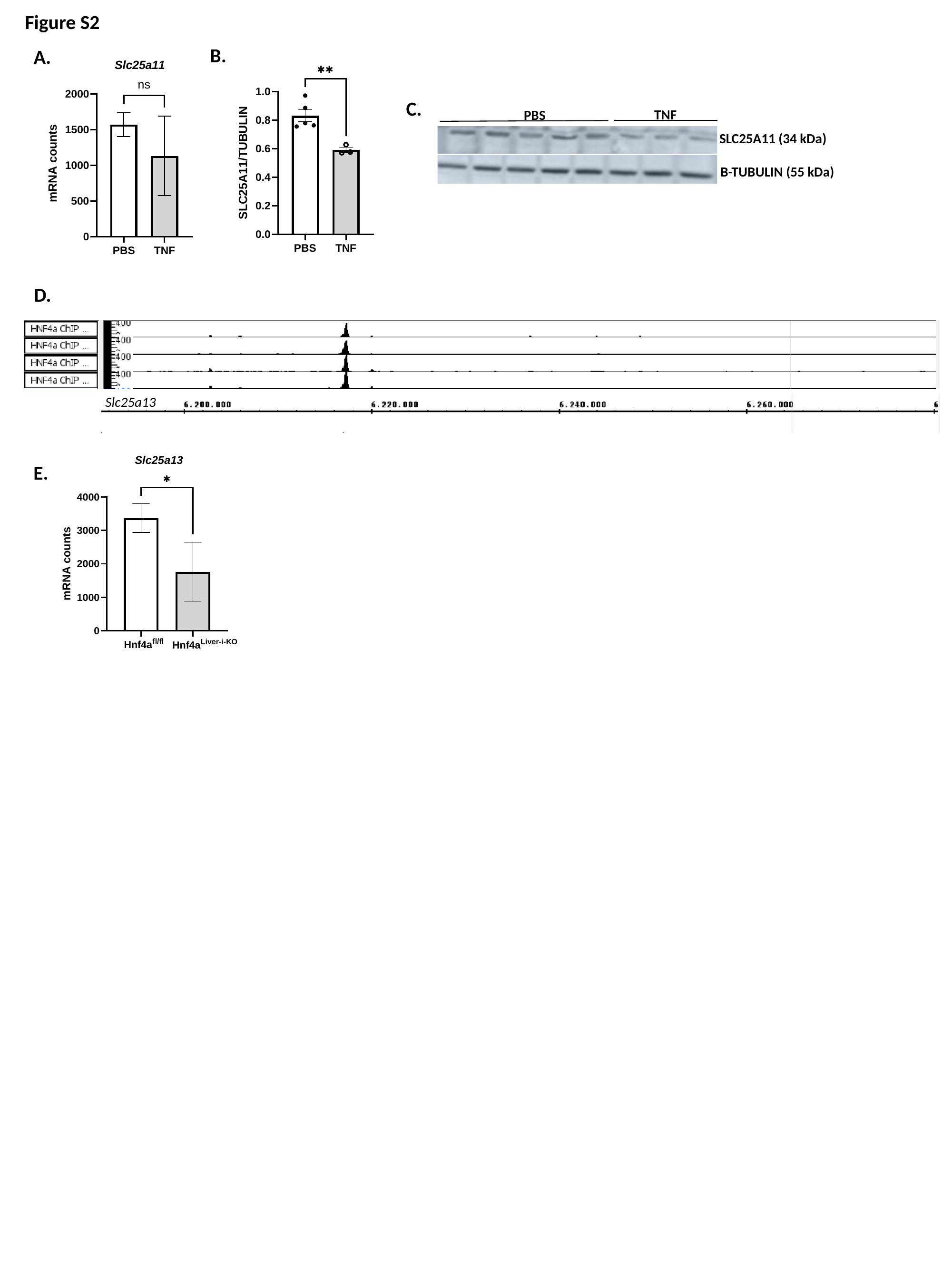

Figure S2
B.
A.
C.
TNF
PBS
SLC25A11 (34 kDa)
B-TUBULIN (55 kDa)
D.
Slc25a13
E.

## Slide 4
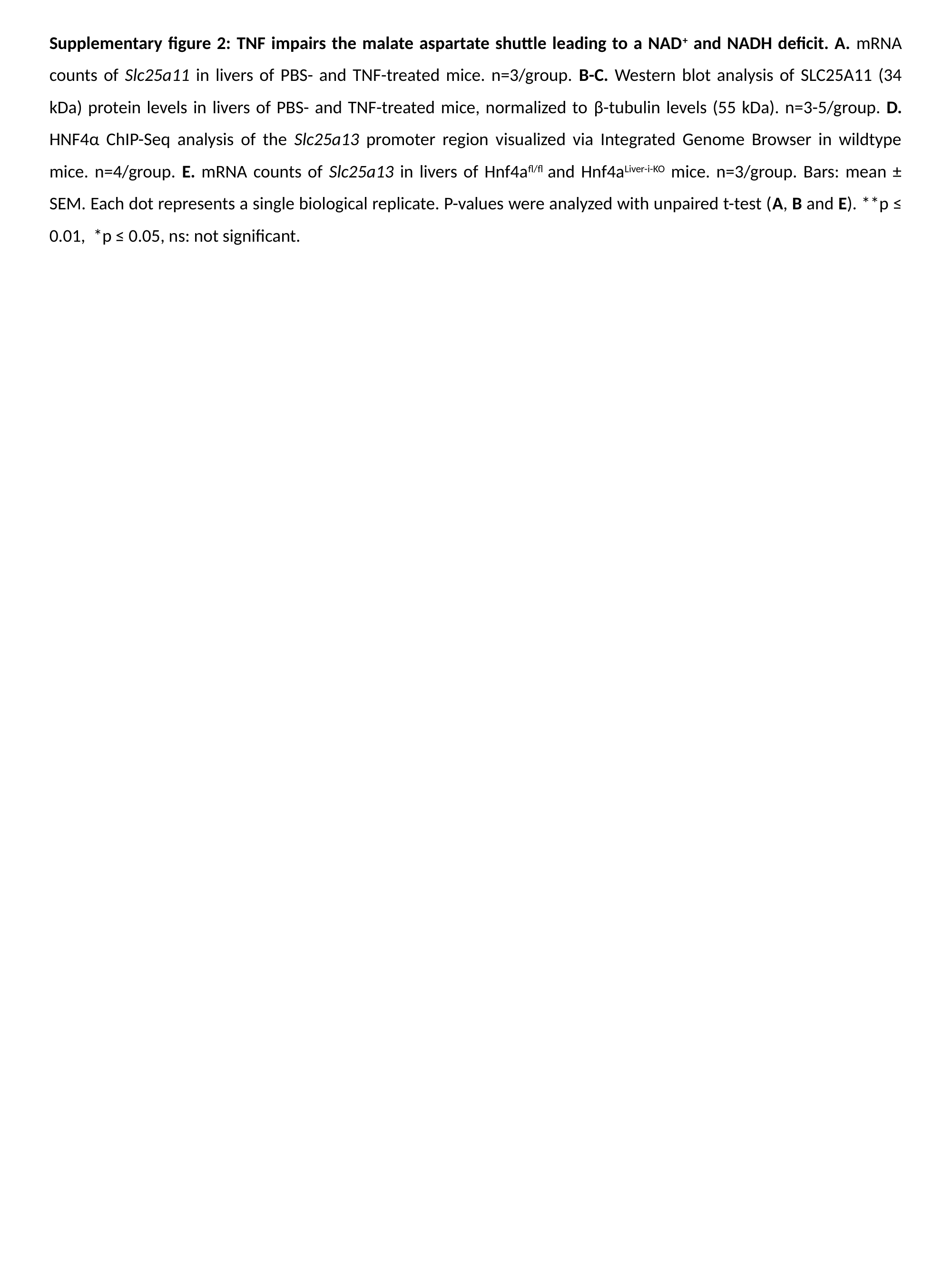

Supplementary figure 2: TNF impairs the malate aspartate shuttle leading to a NAD+ and NADH deficit. A. mRNA counts of Slc25a11 in livers of PBS- and TNF-treated mice. n=3/group. B-C. Western blot analysis of SLC25A11 (34 kDa) protein levels in livers of PBS- and TNF-treated mice, normalized to β-tubulin levels (55 kDa). n=3-5/group. D. HNF4α ChIP-Seq analysis of the Slc25a13 promoter region visualized via Integrated Genome Browser in wildtype mice. n=4/group. E. mRNA counts of Slc25a13 in livers of Hnf4afl/fl and Hnf4aLiver-i-KO mice. n=3/group. Bars: mean ± SEM. Each dot represents a single biological replicate. P-values were analyzed with unpaired t-test (A, B and E). **p ≤ 0.01, *p ≤ 0.05, ns: not significant.

## Slide 5
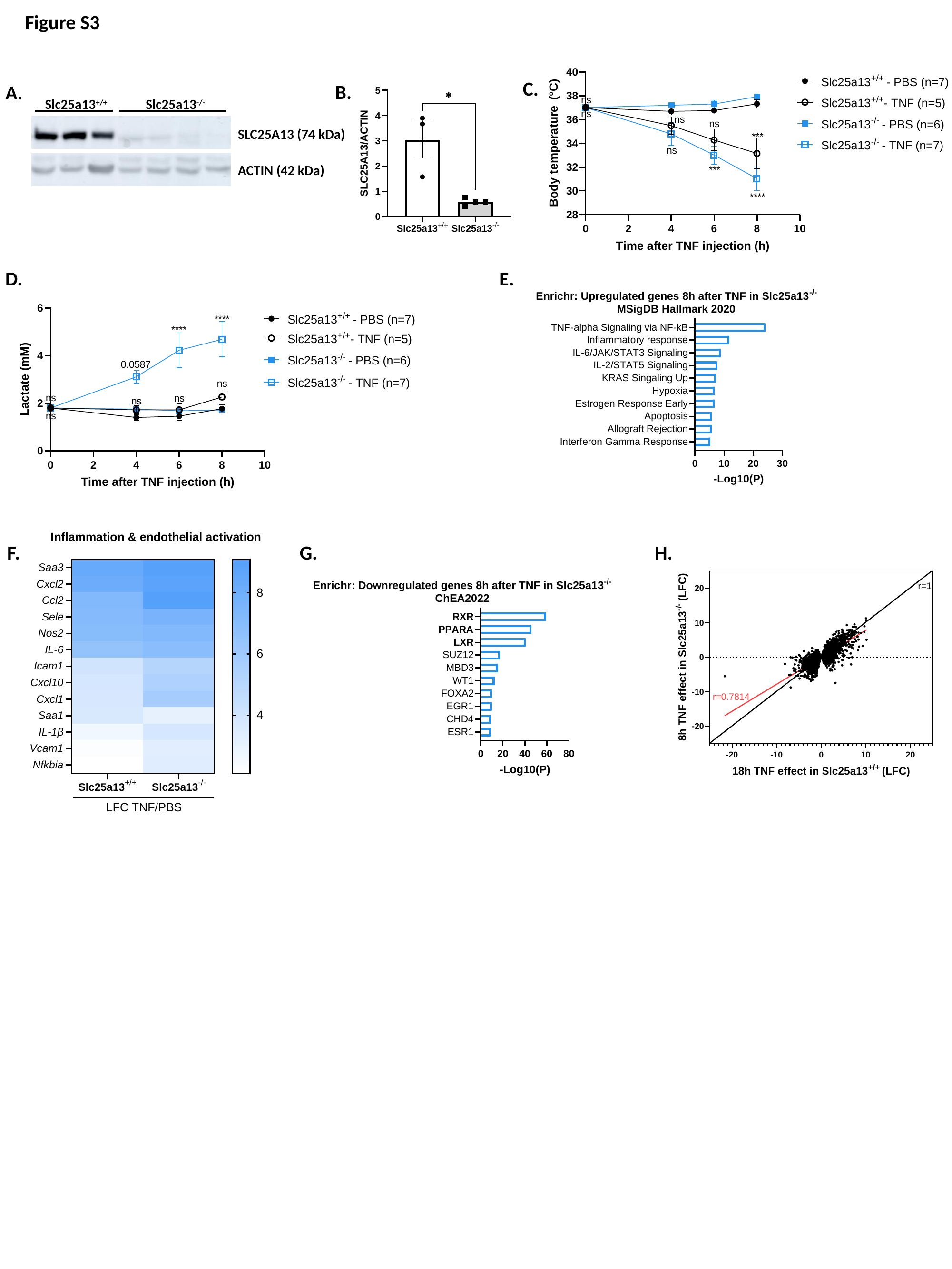

Figure S3
C.
B.
A.
Slc25a13+/+
Slc25a13-/-
SLC25A13 (74 kDa)
ACTIN (42 kDa)
D.
E.
F.
G.
H.

## Slide 6
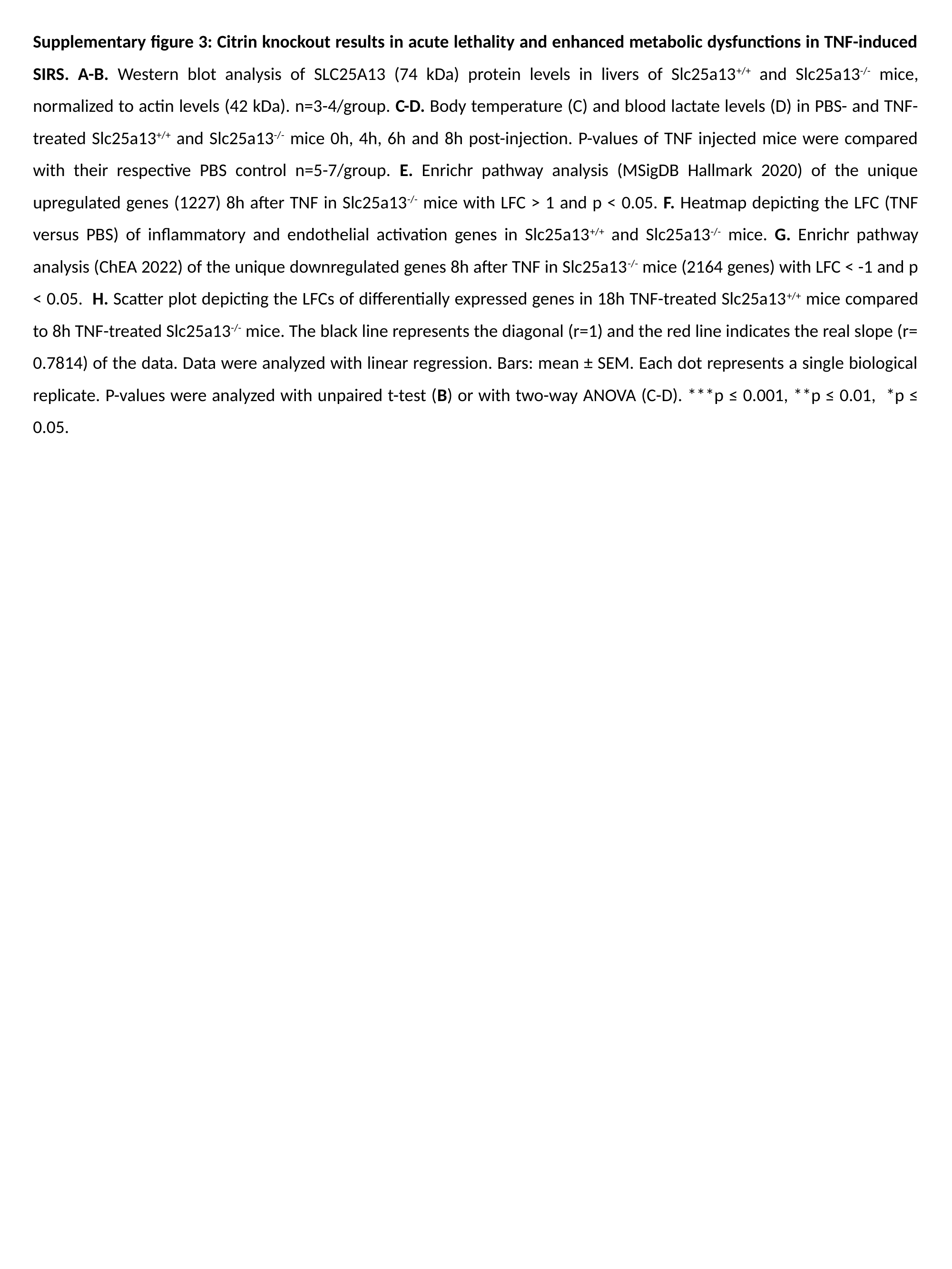

Supplementary figure 3: Citrin knockout results in acute lethality and enhanced metabolic dysfunctions in TNF-induced SIRS. A-B. Western blot analysis of SLC25A13 (74 kDa) protein levels in livers of Slc25a13+/+ and Slc25a13-/- mice, normalized to actin levels (42 kDa). n=3-4/group. C-D. Body temperature (C) and blood lactate levels (D) in PBS- and TNF-treated Slc25a13+/+ and Slc25a13-/- mice 0h, 4h, 6h and 8h post-injection. P-values of TNF injected mice were compared with their respective PBS control n=5-7/group. E. Enrichr pathway analysis (MSigDB Hallmark 2020) of the unique upregulated genes (1227) 8h after TNF in Slc25a13-/- mice with LFC > 1 and p < 0.05. F. Heatmap depicting the LFC (TNF versus PBS) of inflammatory and endothelial activation genes in Slc25a13+/+ and Slc25a13-/- mice. G. Enrichr pathway analysis (ChEA 2022) of the unique downregulated genes 8h after TNF in Slc25a13-/- mice (2164 genes) with LFC < -1 and p < 0.05. H. Scatter plot depicting the LFCs of differentially expressed genes in 18h TNF-treated Slc25a13+/+ mice compared to 8h TNF-treated Slc25a13-/- mice. The black line represents the diagonal (r=1) and the red line indicates the real slope (r= 0.7814) of the data. Data were analyzed with linear regression. Bars: mean ± SEM. Each dot represents a single biological replicate. P-values were analyzed with unpaired t-test (B) or with two-way ANOVA (C-D). ***p ≤ 0.001, **p ≤ 0.01, *p ≤ 0.05.

## Slide 7
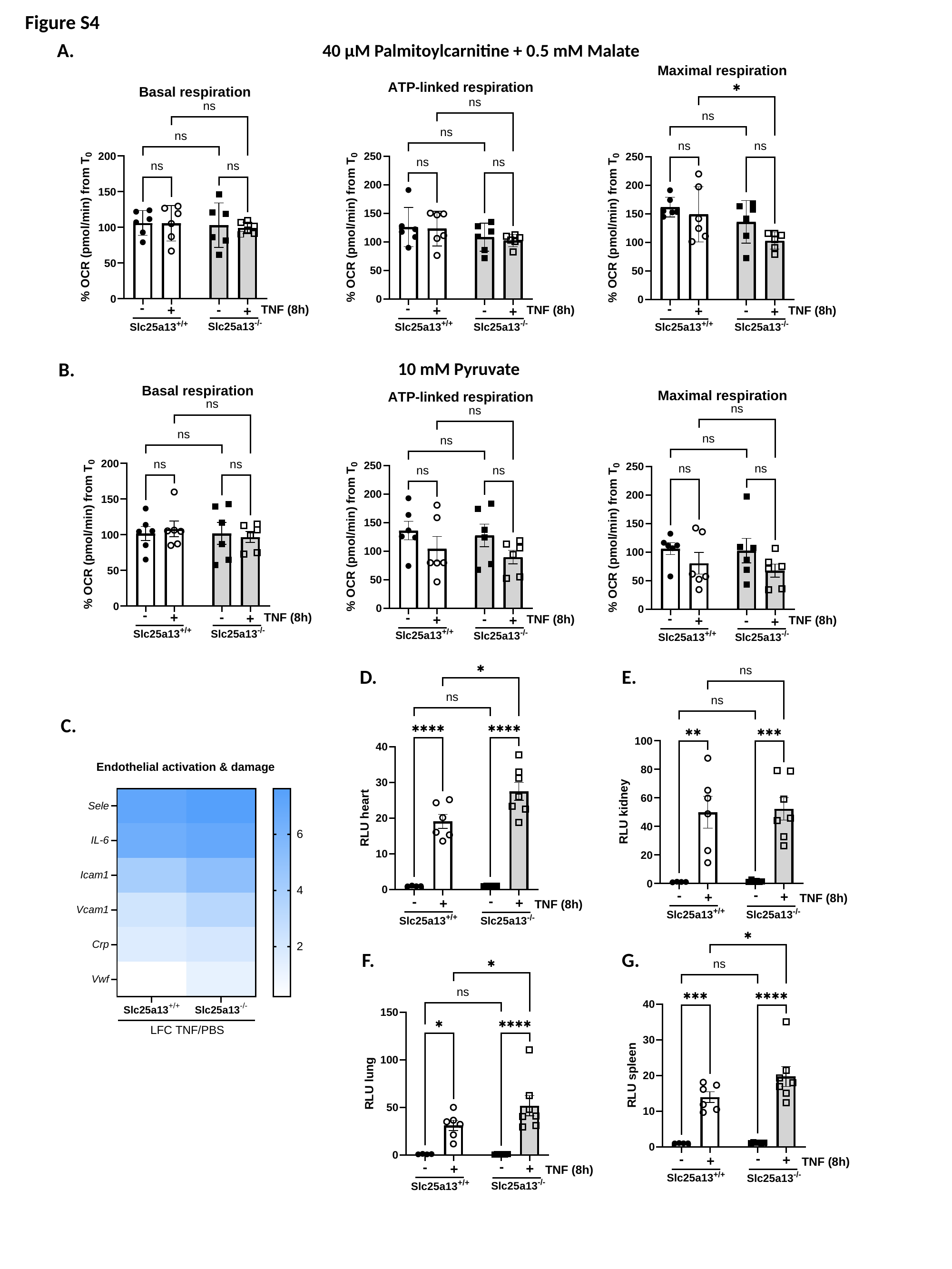

Figure S4
A.
40 µM Palmitoylcarnitine + 0.5 mM Malate
B.
10 mM Pyruvate
D.
E.
C.
F.
G.

## Slide 8
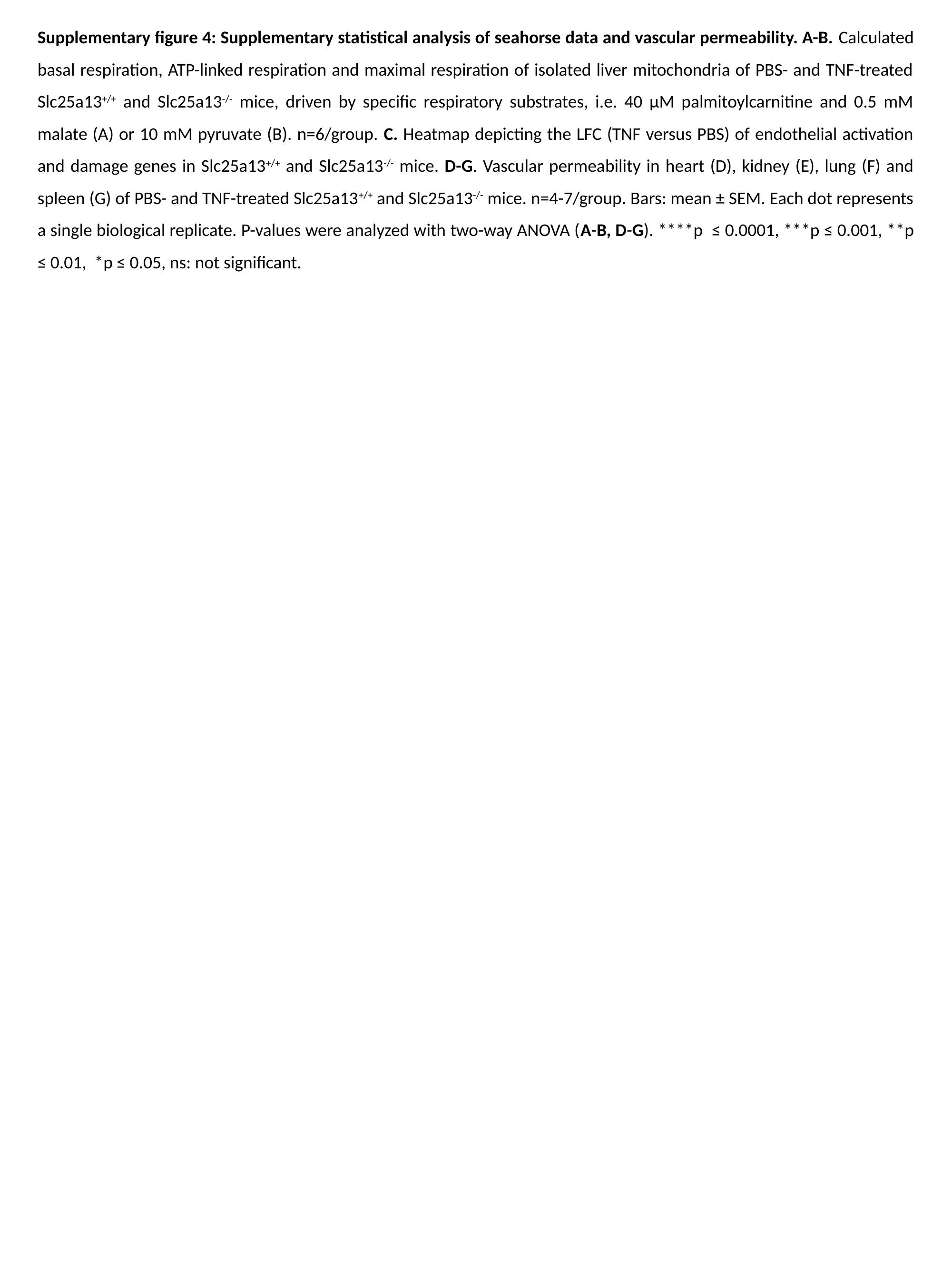

Supplementary figure 4: Supplementary statistical analysis of seahorse data and vascular permeability. A-B. Calculated basal respiration, ATP-linked respiration and maximal respiration of isolated liver mitochondria of PBS- and TNF-treated Slc25a13+/+ and Slc25a13-/- mice, driven by specific respiratory substrates, i.e. 40 µM palmitoylcarnitine and 0.5 mM malate (A) or 10 mM pyruvate (B). n=6/group. C. Heatmap depicting the LFC (TNF versus PBS) of endothelial activation and damage genes in Slc25a13+/+ and Slc25a13-/- mice. D-G. Vascular permeability in heart (D), kidney (E), lung (F) and spleen (G) of PBS- and TNF-treated Slc25a13+/+ and Slc25a13-/- mice. n=4-7/group. Bars: mean ± SEM. Each dot represents a single biological replicate. P-values were analyzed with two-way ANOVA (A-B, D-G). ****p ≤ 0.0001, ***p ≤ 0.001, **p ≤ 0.01, *p ≤ 0.05, ns: not significant.
